# Does Inquiry-Based Learning Improve Students’ Critical Thinking? A Meta-Analysis Accounting for Control Group Variations

**DOI:** 10.12688/f1000research.180569.1

**Published:** 2026-05-14

**Authors:** Abel Harapan, Risky Setiawan, Tri Santi Kurnia, Andini Sakimin, Auliasari Marjan, Sarnus Joni Harto, Israel Ametepey, Fitriana Ibrahim, Alexander Bilaku

**Affiliations:** 1 Educational Research and Evaluation, State University of Yogyakarta Graduate School, Yogyakarta, Special Region of Yogyakarta, Indonesia; 2 Study Program of Biology Education, Faculty of Education and Teachers Training, UIN Abdul Muthalib Sangadji, Ambon, Maluku, Indonesia; 3 MA in Philosophy of Humanities, University of Leiden, Leiden, Netherlands Antilles; 4 Department of Mathematics, Gadjah Mada University, Yogyakarta, Special Region of Yogyakarta, Indonesia

**Keywords:** inquiry-based learning, critical thinking, meta-analysis, effect size, control group variations

## Abstract

**Background:**

Inquiry learning is widely recognized, through empirical studies, as an appropriate instruction in enhancing students’ critical thinking, yet the results were varied across context. The previous meta-analysis did not include the control group variations as a potential moderator and the studies subject area was limited only to science subjects. Consequently, it is difficult to generalize the effectiveness of IBL in enhancing critical thinking.

This meta-analysis aims to investigate whether inquiry learning is effective in improving the students’ critical thinking skills and examine the moderating roles of each study characteristic.

**Methods:**

The literature search applying the PRISMA protocol 2020 was conducted by utilizing SCOPUS, ERIC, and DOAJ databases. A total of 37 studies from 34 articles, published from 2015 to 2025, were synthesized using a random-effects model with standardized mean difference (SMD).

**Results:**

The analysis revealed that IBL has a large and significant effect on enhancing students’ critical thinking (g = 1.48; 95% CI [1.23, 1.73]). However, substantial heterogeneity was observed (I ^2^ = 83.84%), suggesting variability across contexts. Moderator analyses revealed that the main moderator, control group variations, was statistically significant in moderating the effectiveness of IBL (Qm = 8.08; *p* = .018). Similarly, other moderators were significant; subject area (Qm = 15.21; p = .036), education level (Qm = 7.90; *p* = .049), and country ( *Q _m_* (1) = 4.70; *p* = .03), significantly influenced the effectiveness of inquiry learning.

**Conclusions:**

These results highlighted the strong potential of IBL in improving students’ critical thinking. However, the effectiveness of IBL was relative to the type of group control variations, as well as other study characteristics.

## Introduction

Digital technology has developed rapidly leading to an unprecedented expansion of accessible information, including unverified and misleading content. ^1, 2^ In this context, critical thinking has become an essential competency, which enables individuals to evaluate the credibility of information, identify misinformation, and make informed decisions. ^3^ Empirical evidence revealed that students with stronger critical thinking skills are better in detecting fake news, ^4, 5^ analyzing and evaluating evidence or information critically. ^6^

However, numerous studies reported conversely that many students still struggle to distinguish between reliable and misleading information. ^7, 8^ This issue highlights the urgent need for teachers to strengthen students’ critical thinking in learning. ^9^ In general, teachers addressed this problem by applying inquiry-based learning (IBL) that has been widely promoted as an instructional approach. The reason is that IBL actively engages students in questioning, investigating, analyzing evidence, and constructing knowledge. ^10^

However, despite its advantages, the implementation of IBL in classroom practice is not without challenges. Teachers often lack the pedagogical competency, experience necessary to implement IBL effectively, confidence, as well as self-efficacy. ^11^ Moreover, teachers tend to apply conventional or traditional methods rather than IBL, ^12^ difficult in managing individual differences, and have limited resources and time to implement IBL. ^13^ These findings compel teachers’ endeavor to upgrade competency and skills in order to implement inquiry learning effectively in the future.

The empirical studies revealed that IBL can enhance students’ critical thinking skills across various educational levels, from elementary school to higher education students. ^14– 18^ Although numerous studies have examined the effectiveness of IBL in enhancing critical thinking, the findings remain inconsistent across different contexts, educational levels, and subject areas, teaching strategies, learning media, learning duration, and learning evaluation. ^19– 21^ Previous meta-analyses have proved that these characteristics act as moderator variables that cause the heterogeneity of effect size of inquiry learning on students’ critical thinking.

However, there are still some gaps. First, previous meta-analyses predominantly focus on STEM disciplines, ^22^ particularly science subjects. ^19, 23^ Consequently, non-STEM domains such as social sciences, language, culture and arts are underexplored. As a result, the generalizability of findings across broader educational contexts remains unclear.

Second, previous meta-analyses have often overlooked the role of control group variations, even 80% of educational meta-analyses did not include it as a potential moderator. ^24^ In experimental studies, control group conditions vary substantially, such as teacher-centered approaches (i.e., traditional and conventional instruction) or even other student-centered approaches (i.e., problem based learning and STAD). Empirical evidence revealed that the effectiveness of an intervention is inherently relative to the comparison approaches applied. ^25^ Moreover, studies have shown that neglecting variation instructions in control groups may bias effect size estimates and lead to misleading conclusions. ^26^ Despite its importance, this factor has rarely been systematically examined as a moderator in prior meta-analyses.

In addition, prior meta-analyses exhibit methodological limitations related to the analysis and reporting of moderator effects. Some studies provide limited information on the statistical significance of moderators, ^19^ while others report substantial heterogeneity without conducting moderator analyses to explain the observed variation in effect sizes. ^27^ Thus, these limitations may reduce the interpretability and robustness of meta-analytic findings.

These gaps suggest that there is a need for a more comprehensive and methodologically rigorous meta-analysis. Therefore, the present study aims not only to examine the effectiveness of IBL on improving students’ critical thinking, but also to advance previous research by incorporating control group intervention as a key moderator, as well as broaden the subject area of studies. By addressing these limitations, this study hoped to provide more valid and generalizable estimates of the effectiveness of inquiry learning while considering the educational contexts. Specifically, the objectives of the present meta-analysis are to:

1. Estimate the pooled effect size of inquiry-based learning (IBL) on students’ critical thinking skills.
2. Investigate whether the effectiveness of IBL is moderated by study characteristics, including control group variations, country, education level, and subject area.

## Hypothesis

Based on the research objectives above, the hypotheses in this meta-analysis are formulated as follows.

Hypotheses for the first research objective:

H01: Inquiry based learning has no positive and significant effect on students’ critical thinking.

Ha1: Inquiry based learning has a positive and significant effect on students’ critical thinking.

Hypotheses for the second research objective:

H02: The effectiveness of inquiry based learning on students’ critical thinking is not moderated by moderator variables.

Ha2: The effectiveness of inquiry based learning on students’ critical thinking is moderated by the moderator variables.

## Method

### Research design

The research approach used in this study was a quantitative method integrated with meta-analysis. This method was chosen because it aligns with the objective of this research, which is to analyze the effect size of Inquiry-Based Learning (IBL) on students’ critical thinking skills. The PRISMA protocol was applied in the literature search to ensure the transparency and credibility of this meta-analysis. ^28^

### Data analysis

Random effects were applied as the model effect size in this meta-analysis. Since all studies included in this meta-analysis employed experimental designs with contrast groups, the effect size can be estimated using Standardized Mean Difference (SMD). SMD is estimated by dividing the difference between the means in the contrast groups by the pooled standard deviation. ^29^ Furthermore, all studies included in this meta-analysis had different scales. Therefore, SMD was applied to equate the different scales or units of all studies included. ^30, 31^

Among the 34 studies analyzed in this study, three studies did not report the standard deviation for either the experimental group or the control group. These missing data can be addressed by contacting the writer or checking the attachment of the studies. ^32^ The researcher had emailed them, but there was no response until the data analysis stage. The alternative solution taken was to calculate the pooled standard deviation using the available statistical data in the study such as *t*-test, *z*-value, and Mean Squared Error. ^29, 33^

In this meta-analysis, the *metafor* package, developed by (Viechtbauer, 2010), in the R program (2025.09.1 + 401) was used to estimate the individual and pooled effect size and its variance, as well as the standard deviation error, and detect the publication bias. Whereas, JASP (0.96.0.0) was used to analyze subgroup moderators and produce the forest plot. The analysis was conducted using the restricted maximum likelihood (REML) method, with the Knapp–Hartung (KNHA) adjustment to enhance the precision of standard errors and confidence intervals. The effect size of the studies is then interpreted based on the criteria presented in Table 1 below.

**Table 1.** Criteria for interpreting Hedges’ g effect sizes, classifying effect magnitudes from ignored to very large.

Effect size ( *g*)	Interpretation
0.00 *g* < 0,20	Ignored
0,20 ≤ *g* < 0.50	Small
0,50 ≤ *g* < 0.80	Moderate
0,80 ≤ *g* < 1.30	Large
1,30 ≤ *g*	Very large

Since Cohen’s *d* tends to provide a biased population effect size for small samples, ^34^ Hedges’ g was employed due to its bias-correction factor. ^29^ Moreover, Hedges’ *g* provides a more accurate estimate of how much the IBL approach affects students’ critical thinking skills compared to the other methods. ^19^

Heterogeneity of the studies was assessed by Cochran’s *Q* statistic. However, it has limited power to detect true heterogeneity when the number of included studies is small and tends to overestimate when the number of studies is large. ^32^ Therefore, the *I ^2^* statistic is recommended because it is less dependent on the number of studies and offers a clearer and intuitive interpretation of heterogeneity. ^32, 35^ The tentative categorization values of I ^2^ according to Higgins et al. (2003) are 25% (low), 50% (moderate), and 75% (high).

## Literature collection

**Figure 1.**
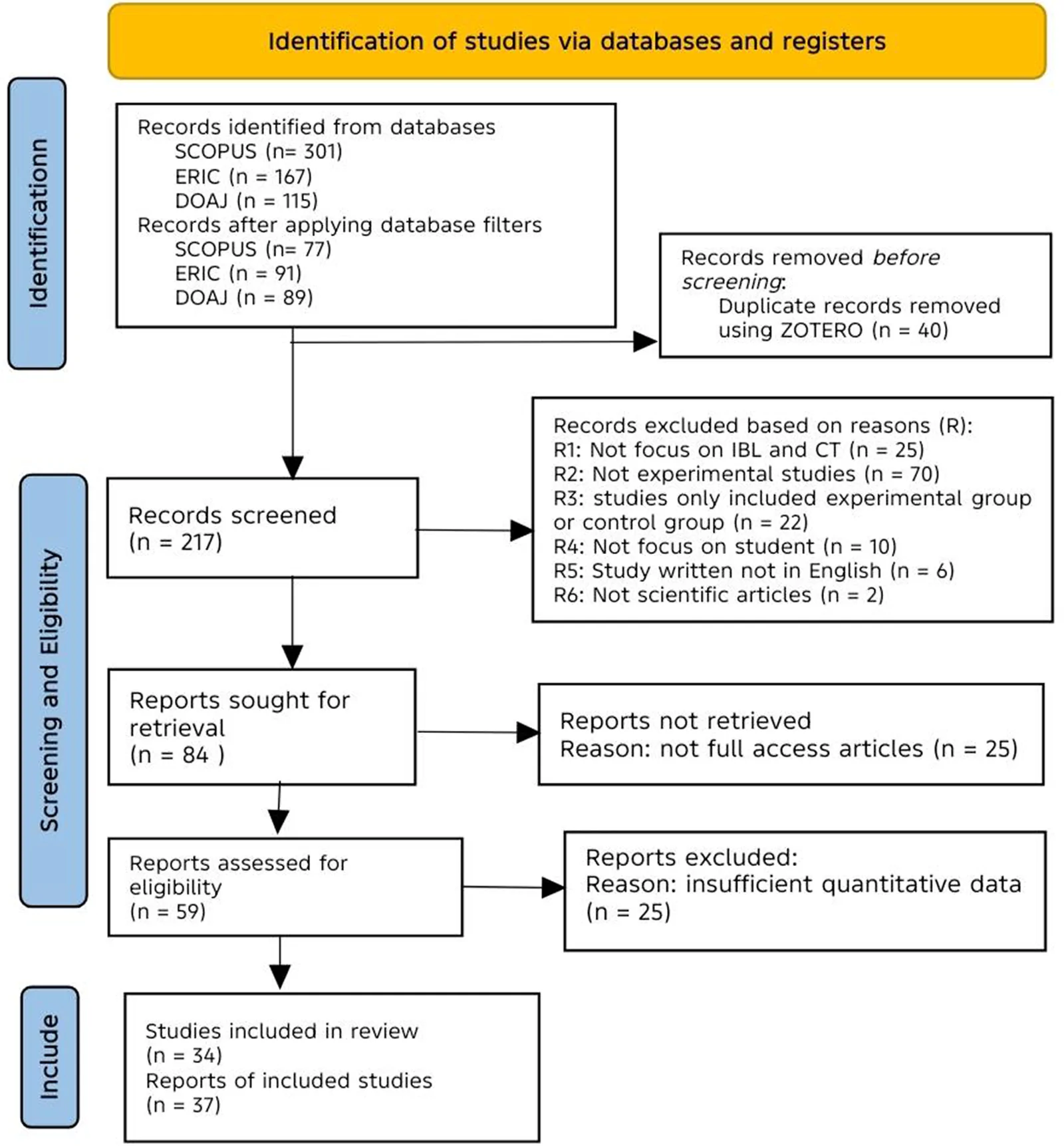
PRISMA flow diagram of the study selection process, showing the number of records identified, screened, excluded, and included, along with reasons (represented by R1-R6) for exclusion at each stage.

The stages of literature collection, which consists of identification, screening and eligibility, and included, are presented in Figure 1.

### Identification

This study began with a systematic collection of relevant literature as data sources through SCOPUS, ERIC, and DOAJ databases. SCOPUS was selected because it provides credible and high-quality articles. ERIC is one of the most prominent databases for meta-analysis because it provides a wide range of literature in the fields of social and educational sciences, ^36, 37^ whereas DOAJ was considered because it provides full access to high-quality literature.

The literature search was done using the keywords combined with Boolean Operators (AND, OR) to ensure comprehensive coverage of relevant literature as well as enhance search precision. ^34, 38^ The search was conducted using the same keywords: “inquiry-based learning” AND “student critical thinking”.

A number of articles identified in the three databases were narrowed using specific filters, such as publication year, subject area, document type, and language (for SCOPUS); publication date, publication type, and peer-reviewed status (for ERIC); subject and publication year (for DOAJ). The remaining articles are displayed in the PRISMA diagram below. The metadata of literature was then imported into ZOTERO in RIS format to remove duplicate articles.

### Screening and eligibility

The remaining articles were screened based on the inclusion and exclusion criteria presented in Table 2.

**Table 2.** Inclusion and exclusion criteria applied in the study selection process.

Inclusion	Exclusion
True/quasi-experimental design	Pre-experimental or qualitative design
Focus on IBL and CT	Not focused on IBL and CT
Participants are from elementary to higher education students	Non-student populations or outside specified levels
Sufficient quantitative data	Insufficient quantitative data
Published between 2015–2025	Published before 2015

Based on Figure 1, ten studies were excluded because their subjects were focused on teachers. Although the literature search through the SCOPUS database was limited to English, 6 articles were found written in Indonesian. This happened because DOAJ publishes articles in various languages, including Indonesian.

A total of 84 studies proceeded to the full-text retrieval stage. At this stage, only open-access and downloadable articles were considered. The downloadable articles were read thoroughly to determine the sufficient amount of substantial data. The eligible articles were then extracted into the coding table.

### Included

A total of 34 independent studies were included, yielding 37 study entries because one study (Lue et al., 2020) reported four statistical datasets based on four distinct samples.

### Data coding

The substantial data, extracted from post-test data of both experimental and control groups of each study, ^39^ were tabulated into the coding table, which consists of the number of samples, mean, and standard deviation of experimental and control groups. ^21, 36^ In addition, moderator variables such as control group variations, subject area, country and education level were also coded. ^29^ The coding procedures were conducted by the first author under the direct supervision of the second author to ensure their accuracy and reliability. ^40^

## Findings and discussion

### Descriptive

The characteristic of each study is presented in Table 3. Overall, the studies involved a total of 2.324 students consisting of 438 elementary school students, 439 junior high school students, 1.105 senior high school students, and 342 higher education students. Based on the group, there are 1.164 students in the experimental group and 1.160 students in the control group.

**Table 3.** Study characteristics. Summary of included studies and moderator variables. One study (Lu et al., 2020) reported four effect sizes, indicated by suffixes (a–d).

No.	Study	N	Effect sizes	Moderator variables			
				Subject	Country	Grade	Type of control group
1	Wulandari et al (2022)	164	1,5317	Biology	Indonesia	Senior High School	Traditional
2	Gunawan et al (2019)	64	1,4780	Physics	Indonesia	Senior High School	Conventional
3	Rahmi et al (2019)	64	1,0440	Biology	Indonesia	Junior High School	Conventional
4	Astina et al (2025)	36	2,1728	Economics	Indonesia	Senior High School	Conventional
5	Mbhanyisi et al (2025)	46	1,2135	Biology	South Africa	Senior High School	Traditional
6	Maharani et al (2023)	53	2,1941	Physics	Indonesia	Elementary school	STAD
7	Anjarwani et al (2020)	64	1,0722	Natural Science learning	Indonesia	Elementary school	Traditional
8	Widarti et al (2024)	72	0,9591	Chemistry	Indonesia	Senior High School	Traditional
9	Latifah & Suprihatiningrum (2024)	40	0,7535	Chemistry	Indonesia	Senior High School	STAD
10	Ghaemi & Mirsaeed (2017)	56	1,9395	English learning	Iran	Higher education	Traditional
11	Arsal (2017)	38	0,2321	Physicology	Turkey	Higher education	Traditional
12	Farah & Ayoubi (2020)	53	2,8142	Chemistry	Lebanon	Senior High School	Traditional
13	Lu et al (2020)a	53	0,5383	Chemistry	Taiwan	Elementary school	Traditional
14	Lu et al (2020)b	58	1,0421	Chemistry	Taiwan	Junior High School	Traditional
15	Lu et al (2020)c	58	1,1040	Chemistry	Taiwan	Elementary school	Traditional
16	Lu et al (2020)d	65	1,6374	Chemistry	Taiwan	Junior High School	Traditional
17	Subagiyo et al (2023)	80	1,8984	Physics	Indonesia	Senior High School	Conventional
18	Syafaren et al (2019)	54	1,4224	Natural Science learning	Indonesia	Junior High School	Conventional
19	Mayarni et al (2023)	50	2,7288	Biology	Indonesia	Elementary school	Conventional
20	Styawan & Arty (2020)	60	0,7547	Chemistry	Indonesia	Senior High School	Problem-Based Learning
21	Lestari & Anggraini (2021)	42	1,9656	English learning	Indonesia	Junior High School	Expository
22	Azizah & Umah (2025)	72	2,3396	Arts and Culture	Indonesia	Elementary school	Conventional
23	Khasawneh et al (2022)	41	1,2319	Mathematics	America	Higher education	Traditional
24	Yue et al (2023)	57	0,6198	English learning	China	Higher education	Communicative Language Teaching (CLT)
25	Carracedo (2025)	54	0,7286	English learning	Spain	Higher education	Traditional
26	Pahrudin et al (2021)	50	1,2037	Physics	Indonesia	Senior High School	STAD
27	Pursitasari et al (2020)	56	1,7689	Natural Science learning	Indonesia	Junior High School	Not reported
28	Gombo (2025)	60	1,7220	Mathematics	Indonesia	Senior High School	Conventional
29	Ritli & Adlini (2022)	52	3,8354	Biology	Indonesia	Senior High School	Conventional
30	Sholikhan & Kusnadi (2021)	128	1,2665	Physics	Indonesia	Senior High School	Conventional
31	Damayanti (2025)	72	3,0755	Economics	Indonesia	Senior High School	Conventional
32	Sucilestari & Arizona	61	2,2320	Physics	Indonesia	Senior High School	Cooperative learning
33	Musyawwir et al (2023)	56	1,0836	Natural Science learning	Indonesia	Elementary school	Not reported
34	Kitot et al (2015)	83	1,5735	History	Malaysia	Senior High School	Traditional
35	Purwanita et al (2019)	50	1,3248	History	Indonesia	Elementary school	Conventional
36	Aido et al (2022)	94	0,8198	Chemistry	Ghana	Higher education	Traditional
37	Nurhalisa & Rahmawaty (2025)	68	0,9419	Natural Science learning	Indonesia	Junior High School	Conventional

### Summary effect size

**Figure 2.**
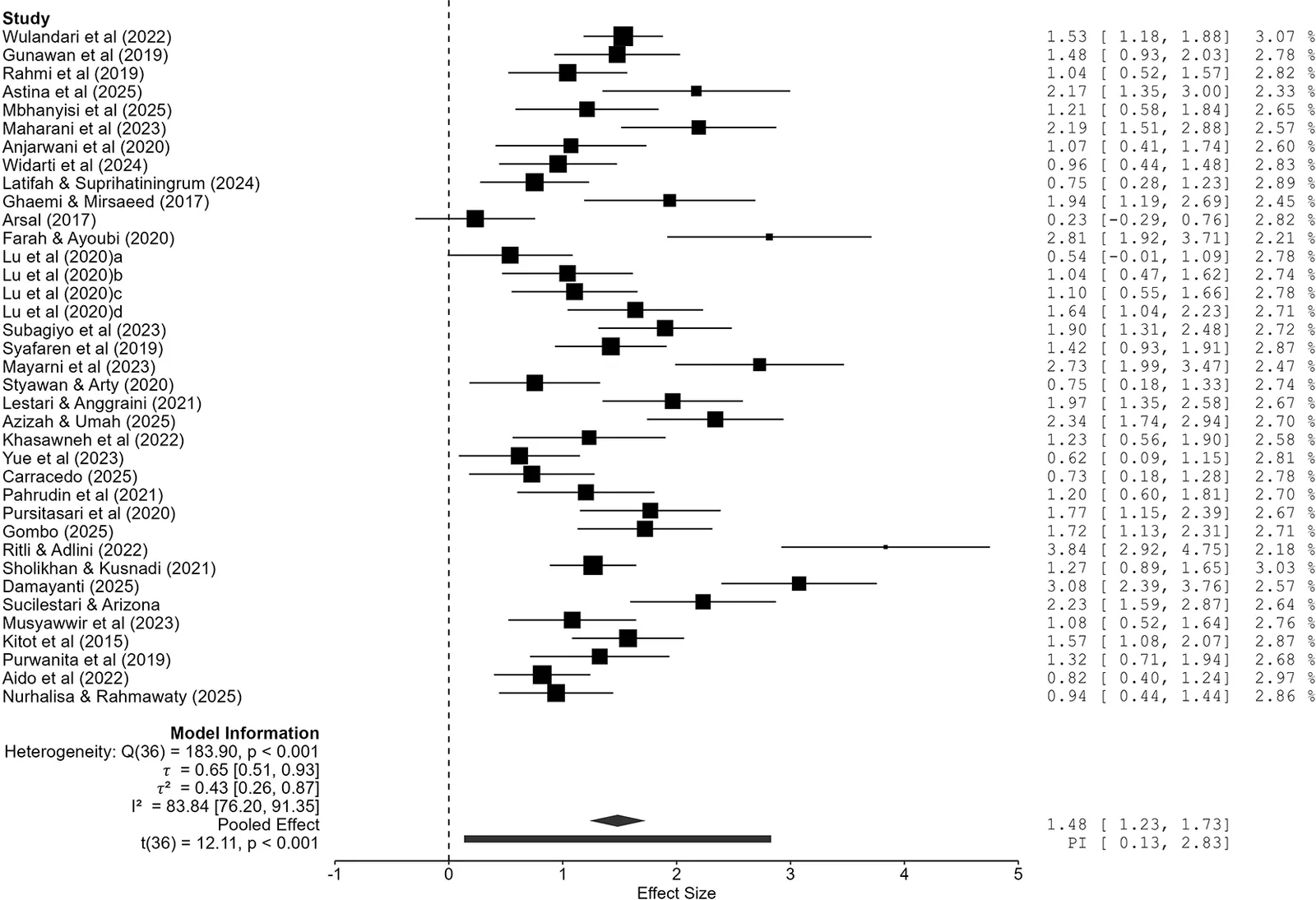
Forest Plot. Forest plot of Hedges’ g for 37 effect sizes derived from 34 studies. Squares represent individual effect sizes (proportional to study weight), horizontal lines indicate 95% confidence intervals, and the diamond represents the pooled effect size.

Figure 2 represents the forest plot that encompasses the substantial statistics data analyzed in this meta-analysis, such as individual effect size along with its study weight and confidence interval (CI), summary effect size, and statistics heterogeneity. It is shown that the summary effect size estimated using a random-effects model is 1.48 with a *p*-value <0,001. Based on Hedge’s *g* criteria, this value indicates that IBL has a large and significant effect on students’ critical thinking (CT).

The forest plot also demonstrates that all studies included in this meta-analysis have positive and statistically significant effects, as indicated by confidence intervals (CI) 95% ranged from 1.23 to 1.73 that do not cross the line of zero. ^19, 41^ However, studies by Ghaemi & Mirsaeed (2017) and Lu et al. (2020) show insignificant effect, as indicated by the CI that crosses the zero line.

The summary effect size is based on weighted individual effect sizes of the whole studies, with effect sizes from larger samples weighted more than the effect sizes from smaller samples. ^41^ A study by Wulandari et al. (2022) has the largest contribution to the summary effect size, indicated by the highest study weight of 3.07%. Conversely, the study by Ritli & Andlini (2022) gives the lowest contribution indicated by the lowest study weight (2.18%).

The heterogeneity analysis indicated substantial variability among the included studies. ^19, 34^ This was shown by Cochran’s *Q* and I ^2^ tests. Cochran’s *Q* test shows statistically significant heterogeneity *Q* (36) = 179.64, p < 0.001. It means that the observed variability in effect sizes cannot be explained solely by sampling error. ^30^ Similarly, the high value of the I ^2^ statistic shows that 83.84% of the total variability in effect sizes was caused by real differences between studies rather than random variation. Collectively, these statistics consistently indicate substantial heterogeneity and the subsequent exploration of potential moderator variables. ^42^

The findings of this meta-analysis show positive and significant summary effects of the thirty-seven studies regarding the effect of inquiry-based learning on students’ critical thinking skills, with a Hedge’s g = 1.48. Based on Cohen’s d criteria, this effect is classified as a very large effect. It means that students who engage in IBL demonstrate substantially higher critical thinking skills compared to those receiving traditional or other learning models applied in the thirty-seven studies. This finding confirms that IBL can be a powerful learning model in enhancing student’s critical thinking skills.

This result is larger than that of the previous meta-analysis by Arifin et al. (2025) ^19^ (g (N = 36) = 1.27; 95% CI [0.78, 1.76]). Despite showing large and significant effects, their meta-analysis was limited only on studies conducted on natural science subjects. In contrast, our meta-analysis covered the studies in the field of natural science, mathematics, social science, language, as well as arts and culture. Therefore, with the large summary effect size, it can be inferred that IBL is not only effective in natural science learning, but also in mathematics, social science and language. Nevertheless, this meta-analysis confirms the meta-analysis by Arifin et al. (2025) ^19^ in terms of summary effect and confidence interval. It is shown that the summary effect size of this meta-analysis is still in the range of confidence interval of pooled effect size of their meta-analysis. Therefore, the present meta-analysis is consistent with the previous meta-analysis.

The strong effect of IBL on critical thinking can be explained through its core pedagogical characteristics. Unlike traditional methods, which often emphasize the transmission of factual knowledge, inquiry based learning engages students in some activities enhancing students critical thinking, such as constructing knowledge, explaining, reasoning, questioning, and communicating with their instructor or their peers. ^43^ These activities are the substantial component of critical thinking. Overall, the results suggest that teachers should apply IBL in the classroom in order to enhance students’ critical thinking.

Despite a large and significant pooled effect, the individual studies included in this meta-analysis showed considerable variation in effect sizes. For instance, a study by Arsal (2017) shows negligible effect (g = 0.2321; 95% CI [−0.29,0.76]). This finding indicates that the effectiveness of IBL on critical thinking may depend on some factors acting as the moderator variables. Thus, this meta-analysis not only analysed the overall effect size, but also detected the potential moderator variables that are responsible for the variety of effect size across the studies, including level of education, subject, and country.

### Moderator analysis

Moderator analysis was conducted to examine the sources of the heterogeneity of effect size, ^41, 44^ that may influence the effectiveness of IBL on critical thinking in this meta-analysis. Since the moderator variables considered in this meta-analysis are categorical moderators, the subgroup analysis was applied to analyze moderator variables. ^45^ Recent meta-analysis complements the methodological limitation of the previous meta-analysis by reporting the significance of the subgroup analysis. The subgroups were divided into four groups, i.e., control group variations, subject area, level of education, and country.

### Control group variations

The subgroup analysis confirmed that in this meta-analysis, variations in the control group acted as a significant moderator, as indicated by the subgroup differences (Q _m_ (2) = 7.25; p = .027). Initially, six subgroups were identified. However, three of them were automatically excluded by the system due to an insufficient number of studies, as a minimum of two studies per subgroup was required for inclusion.

Studies employing traditional methods in the control group demonstrated a large and significant pooled effect (g = 1.187; 95% CI [0.864, 1.509]), followed by those using conventional methods (g = 1.883; 95% CI [1.420, 2.346]). These findings indicate that the effectiveness of inquiry learning is substantially higher when compared to traditional or conventional teaching approaches. In contrast, studies using Student Teams Achievement Division (STAD) in the control group yielded a relatively large effect size (g = 1.278; 95% CI [0.309, 2.246]). However, this subgroup exhibited the lowest level of precision, as reflected by the wide confidence interval. Moreover, the comparative effectiveness of inquiry learning appears to decrease when contrasted with STAD, which is recognized as a form of student-centered learning.

Overall, these findings prove that the effectiveness of IBL on students’ critical thinking depends on the learning approaches applied in the control group. When compared with teacher-centered learning approaches, its effectiveness will be higher. In contrast, its effectiveness will be relatively lower when compared with other student-centered learning approaches.

### Country of study

The studies included in this meta-analysis were conducted across eleven countries. However, nine countries (South Africa, Turkey, Iran, Lebanon, the United States, China, Malaysia, Ghana, and Spain) were automatically excluded from the subgroup analysis due to an insufficient number of studies. Consequently, only two countries – Indonesia and Taiwan – were included in the subgroup analysis. The analysis revealed that studies conducted in Indonesia show a large and statistically significant pooled effect size ( *g* (23) = 1.659; 95% CI [1.346, 1.972]]), indicating a strong effect of inquiry learning on student’s critical thinking skills. Similarly, studies from Taiwan also show a large and significant pooled effect ( *g* (3) = 1.071; 95% CI [0.359, 1.784]). The findings reveal that the effectiveness of inquiry learning is relatively inconsistent across countries.

The possible reasons for these results are the differences in pedagogical approaches, the quality of teacher training, or even cultural differences in learning can affect outcomes. ^19^ In Taiwan, students tend to exhibit tutor-oriented and collectivistic learning behaviors, which may moderate the impact of inquiry learning interventions. ^46^ Whereas, in Indonesia, there is a stronger emphasis on response efficacy and performance expectancy in adopting new instructional innovations, which could amplify the perceived or actual effectiveness of inquiry learning interventions. ^47^ The difference effect size between countries is significant, as shown by the *Q _m_* (1) = 4.72; *p* = 0.03, meaning that the country may influence the effectiveness of inquiry learning on critical thinking, as well as explain the heterogeneity in this meta-analysis. Nevertheless, this finding should be interpreted with caution, as it is based on a limited number of studies within each subgroup and therefore cannot be generalized to broader contexts. ^44^

### Domain subject

The studies included in this meta-analysis were focused on ten subjects. However, two of them were excluded automatically by the system because of an insufficient number of studies. Overall, the analysis showed statistically significant heterogeneity with Q _m_ (7) = 15.21; p = 0.036, meaning that the variety in subjects could contribute to the heterogeneity of effect sizes. Hence, the subject domain is a potential moderator influencing the effect of inquiry learning on critical thinking.

Specifically, studies in biology ( *g* = 2.026; 95% CI [0.580, 3.472]) and physics ( *g* = 1.672; 95% CI [1.196, 2.147]) show the very large and significant pooled effect sizes. Similarly, chemistry, natural science, and english learning have significant and moderate effect, i.e, (g = 1.092; 95% CI [0.617, 1.568]), (g = 1.248; 95% CI [0.841, 1.654]), and (g = 1.287; 95% CI [0.110, 2.463]). In contrast, despite their large effect, studies in economics, mathematics, and history are statistically insignificant, as indicated by their CI that crosses the zero line. Studies in economics have *g* = 2.655; 95% CI [−3.066, 8.376], and studies in mathematics have *g* = 1.504, 95% CI [−1.592, 4.599]. Whereas, studies in history have a smaller but more precise effect size than studies in economics and in mathematics ( *g* = 1.476, 95% CI [−0.067, 3.019]).

Note that studies in biology show the largest effect, which confirms the previous meta-analysis by Arifin et al. (2025). A possible explanation for these results is that biology inherently involves processes such as observation, experimentation, and interpretation of natural phenomena, which align well with inquiry learning. ^48– 50^ These processes engage students in higher-order cognitive activities, such as analysis, inference, and causal reasoning, and evaluation, which are core aspects in developing critical thinking. ^20, 51^

Furthermore, studies in the field of science (physics, biology, and chemistry) show a larger effect than that of social science, mathematics, and language. The implementation of inquiry learning in social studies is not fully consistent because when teachers use structured frameworks such as the Inquiry Design Model (IDM), they tend to adjust and modify these approaches to fit their students and classroom conditions. ^52^ As a result, students are not fully engaged in inquiry processes. The other study found that the effectiveness of inquiry learning on the concrete subjects will be more effective than that on abstract subjects, such as mathematics. ^53^

### Education grade

The subjects of the studies analyzed in this meta-analysis were distributed into four levels of education – from elementary school to higher education. The analysis approved that level of education is statistically significant with differences between group Q _m_ (3) = 7.90; *p* = 0.049, indicating that this moderator has a significant effect on moderating the effectiveness of IBL on students’ critical thinking.

Specifically, studies conducted in senior high school, junior high school, and elementary school have very large and significant pooled effect sizes, as shown by following statistics (representing the three grade, respectively), g (15) = 1.731, 95% CI [1.286, 2.177], g (6) = 1.378; 95% CI [1.010, 1.746], and g (7) = 1.529; 95% CI [0.890, 2.168]. In contrast, despite significance, studies conducted in higher education showed a moderate pooled effect, as indicated by g (5) = 0.882; 95% CI [0.293, 1.471].

At senior high school level (g = 1,728), inquiry learning shows a larger effect on students’ critical thinking than other education levels. This result contradicts the meta-analysis by (Arifin et al., 2025) whose result shows that the effect of inquiry learning on students’ critical thinking at postgraduate level is the largest (g = 2.66). Therefore, it can be concluded that the effectiveness of inquiry learning on students’ in higher education is not always larger than that on lower education. It depends on the inquiry model applied. Research based on the PISA 2015 dataset demonstrates that different types of inquiry-based learning can relate differently to student learning outcomes. ^54^ Specifically, guided inquiry was positively associated with science literacy scores, whereas open inquiry was found to have a negative relationship with science literacy in the same models. Thus, it is possible that the studies analyzed in this meta-analysis conducted in senior high school were applying the guided inquiry. Whereas, the studies conducted in higher education were applying open inquiry.

Overall, subgroup analysis revealed that the three moderator variables analyzed in this meta-analysis were statistically significant, which means that these moderator variables were responsible for the heterogeneity of effect size in this meta-analysis. In other words, the effectiveness of Inquiry learning in enhancing students’ critical thinking is influenced by these moderator variables.

### Publication bias

**Figure 3.**
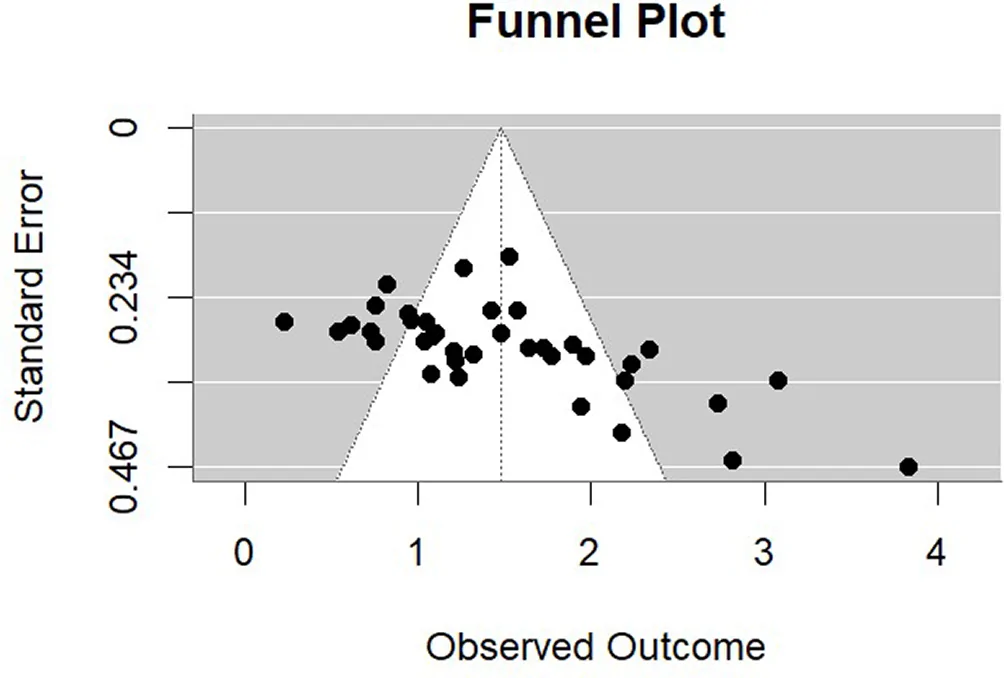
Funnel plot of standardized mean differences (Hedges’ g) for the included studies. The distribution of effect sizes is asymmetrical, suggesting substantial evidence of publication bias.

In the absence of publication bias, the estimated individual effect sizes tend to distribute symmetrically on both sides around the pooled effect size. ^29^ The funnel plot in Figure 3 demonstrates the asymmetrical plot, as indicated by the small studies that tend to distribute on the right side. The asymmetrical funnel plot indicates the presence of publication bias in this meta-analysis. However, since the interpretation of funnel plot tends to be subjective, ^55^ Egger’s Regression test is needed as a further analysis to quantify the asymmetry of the funnel plot. ^29^ The *Metafor* package in R was applied to analyze publication bias.

The publication bias analysis showed that all of the methods applied to assess the publication bias were statistically significant, as shown by Table 4 below.

**Table 4.** Results of publication bias assessment, including Fail-Safe N, Kendall’s Tau, and Egger’s regression test.

Test name	value	p
Fail-Safe N	12126.000	<.001
Kendall’s Tau	0.505	<.001
Egger’s Regression	5.177	<.001

Based on Table 4, it is shown that Fail-Safe N shows a very large number, meaning that an extremely high number of unpublished studies with null findings would be required to nullify the observed effect. This suggests that this meta-analysis is statistically robust and not easily overturned by potential missing evidence. ^56^ However, robustness alone does not guarantee the absence of publication bias. Kendall’s Tau suggests a systematic association between effect sizes and their variances. ^57^ In practical terms, this means that studies with lower precision (typically smaller studies) tend to report larger effects. ^58, 59^ This pattern is widely recognized as small-study effects, which are often linked to selective publication or reporting practices in meta-analysis. ^29^

**Figure 4.**
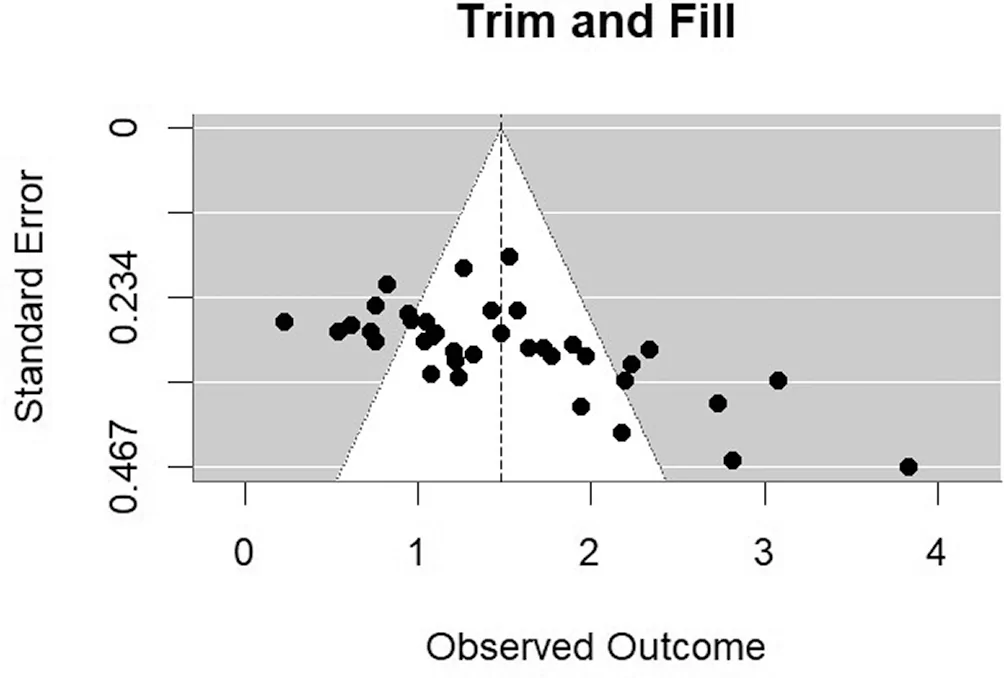
Funnel plot after applying the Trim and Fill procedure. No studies were imputed, and the pooled effect size remained unchanged, supporting the absence of substantial publication bias.

The asymmetrical funnel plot is confirmed by the Egger’s regression test. A significant result in Egger’s test indicates asymmetry in the funnel plot, suggesting that smaller studies yield systematically larger effect sizes than expected under a symmetric distribution. ^60^ Therefore, Egger’s test indicates the presence of publication bias in this meta-analysis. Since the Egger’s test was statistically significant, the analysis was processed to trim and fill the test to identify the missing studies. ^55^ The analysis was done by applying the *metafor* package in R. The trim and fill test showed that there were no missing studies found in this meta-analysis, as shown visually in Figure 4. After the trim and fill process, the pooled effect size does not change (g = 1.48; p-value < .0001; 95% CI [1.2495, 1.7143]). Moreover, the heterogeneity remains the same (i.e., I ^2^ = 83.84%). Figure 3 and Figure 4 above demonstrate the funnel plot before and after the trim and fill. It is shown clearly that the funnel plot is still the same, which shows visually that there were no missing studies in this meta-analysis.

The asymmetrical funnel plot shows that there is publication bias in this meta-analysis. The Egger’s Regression test confirms statistically the presence of publication bias shown by the funnel plot. Yet, Trim and Fill analysis showed no missing studies. Funnel plot asymmetry and a significant Egger’s test indicate the presence of small-study effects but do not constitute definitive evidence of publication bias. ^61^ Such asymmetry may also arise from between-study heterogeneity, methodological differences, or contextual variations. Moreover, given the limited sensitivity of the trim-and-fill method under heterogeneous conditions, the absence of imputed studies suggests that the observed asymmetry is more likely attributable to study characteristics rather than publication bias alone. ^62^

### Results of hypothesis testing

Regarding the effect of IBL on students’ critical thinking, it is shown that IBL has a large and significant effect on students’ critical thinking. Therefore, the null hypothesis (H01) was rejected and the alternative hypothesis (Ha1) was accepted. Similarly, the moderator testing showed that each of the moderator variables significantly moderated the effectiveness of IBL on students’ critical thinking. Thus, the null hypothesis (H02) was rejected and the alternative hypothesis (Ha2) was accepted.

## Conclusion

Present meta-analysis demonstrates that Inquiry-Based Learning (IBL) has a large and significant positive effect on students’ critical thinking skills. It confirms its effectiveness as a powerful instructional approach across various educational contexts. The results suggest that students engaged in inquiry learning consistently outperform those in traditional, conventional and STAD learning environments in terms of critical thinking development. However, the effectiveness of inquiry learning is not uniform across all contexts. The findings reveal that control group variations, country, subject area, and education level significantly influenced the magnitude of its impact. Inquiry learning as a student-centered approach, tends to be higher when compared to teacher-centered learning (traditional and conventional) and lower when compared with other student-centered approaches (STAD). Moreover, it tends to be more effective in science-related subjects and at the senior high school level, while its effectiveness is relatively lower in higher education and certain abstract disciplines such as mathematics and economics. These variations highlight the importance of contextual and pedagogical factors, including the type of inquiry implemented, such as guided versus open inquiry. Although indications of publication bias were detected, further analysis suggests that the observed asymmetry is more likely influenced by heterogeneity and study characteristics rather than bias alone. Therefore, the findings remain robust but should be interpreted with caution. In conclusion, this study reinforces the importance of implementing well-structured inquiry-based approaches in education, particularly those that are guided and context-sensitive, to maximize their impact on students’ critical thinking skills.

### Future directions

Future research is recommended to explore additional moderating variables and to include more diverse contexts to enhance the generalizability of findings.

## Data Availability

Zenodo. Does Inquiry-Based Learning Improve Students’ Critical Thinking? A Meta-Analysis Accounting for Control Group Variations [Data set]. https://doi.org/10.5281/zenodo.19673866 ^63^ The project contains the following underlying data:
- dataset.xlsx (List of all studies with relevant details for statistical analysis) This project contains the following extended data:
-
Figure 1 – PRISMA Flowchart.tif -
Figure 2 – Forest plot.tiff -
Figure 3 – Funnel plot trim and fill.tiff -
Figure 4 – Funnel plot.tiff - PRISMA 2020 – checklist abstract and review.docx - PRISMA 2020 – abstract checklist.docx - R Script.qmd All data are available under the terms of the Creative Commons Attribution 4.0 International (CC-BY 4.0) license.
