# Does Inquiry-Based Learning Improve Students’ Critical Thinking? A Meta-Analysis Accounting for Control Group Variations

**DOI:** 10.12688/f1000research.180569.2

**Published:** 2026-08-08

**Authors:** Abel Harapan, Risky Setiawan, Tri Santi Kurnia, Andini Sakimin, Auliasari Marjan, Sarnus Joni Harto, Israel Ametepey, Fitriana Ibrahim, Alexander Bilaku

**Affiliations:** 1 Educational Research and Evaluation, State University of Yogyakarta Graduate School, Yogyakarta, Special Region of Yogyakarta, Indonesia; 2 Study Program of Biology Education, Faculty of Education and Teachers Training, UIN Abdul Muthalib Sangadji, Ambon, Maluku, Indonesia; 3 MA in Philosophy of Humanities, University of Leiden, Leiden, Netherlands Antilles; 4 Department of Mathematics, Gadjah Mada University, Yogyakarta, Special Region of Yogyakarta, Indonesia

**Keywords:** inquiry-based learning, critical thinking, meta-analysis, effect size, control group variations

## Abstract

**Background:**

Inquiry learning is widely recognized, through empirical studies, as an appropriate instruction in enhancing students’ critical thinking, yet the results were varied across context. The previous meta-analysis did not include the control group variations as a potential moderator and the studies subject domain was limited only to science subjects. Consequently, it is difficult to generalize the effectiveness of IBL in enhancing critical thinking. This meta-analysis aims to investigate whether inquiry learning is effective in improving the students’ critical thinking skills and examine the moderating roles of each study characteristic. Methods The literature search applying the PRISMA protocol 2020 was conducted by utilizing SCOPUS, ERIC, and DOAJ databases. A total of 57 studies from 51 articles, published from 2015 to 2025, were synthesized using a random-effects model with standardized mean difference (SMD).

**Results:**

The analysis revealed that IBL has a large and significant effect on enhancing students’ critical thinking (g = 1.336; 95% CI [1.061, 1.611]). However, substantial heterogeneity was observed (I ^2^ = 92.09%), suggesting variability across contexts. Moderator analyses revealed that the main moderator, control group variations, was statistically significant in moderating the effectiveness of IBL (Qm = 5.21; *p* = .022). in contrast, subject domain (Qm = 1.43; p = .698), education level (Qm = 1.11; *p* = .774), and country ( *Q _m_* = 3.33; *p* = .650), were insignificantly moderating the effectiveness of inquiry learning.

**Conclusions:**

The present meta-analysis highlighted that IBL is effective in improving students’ critical thinking. However, the effectiveness of IBL was relative to the type of control group variations. Its effect on critical thinking was greater when compared with teacher-centered learning but smaller when compared with other student-centered learning.

## Introduction

Digital technology has developed rapidly leading to an unprecedented expansion of accessible information, including unverified and misleading content. ^1^ ^,^ ^2^ In this context, critical thinking has become an essential competency, which enables individuals to evaluate the credibility of information, identify misinformation, and make informed decisions. ^3^ Empirical evidence revealed that students with stronger critical thinking skills are better in detecting fake news, ^4^ ^,^ ^5^ analyzing and evaluating evidence or information critically. ^6^

However, numerous studies reported conversely that many students still struggle to distinguish between reliable and misleading information. ^7^ ^,^ ^8^ This issue highlights the urgent need for teachers to strengthen students’ critical thinking in learning. ^9^ In general, teachers addressed this problem by applying inquiry-based learning (IBL) that has been widely promoted as an instructional approach. The reason is that IBL actively engages students in questioning, investigating, analyzing evidence, and constructing knowledge. ^10^

However, despite its advantages, the implementation of IBL in classroom practice is not without challenges. Teachers often lack the pedagogical competency, experience necessary to implement IBL effectively, confidence, as well as self-efficacy. ^11^ Moreover, teachers tend to apply conventional or traditional methods rather than IBL, ^12^ difficult in managing individual differences, and have limited resources and time to implement IBL. ^13^ These findings compel teachers’ endeavor to upgrade competency and skills in order to implement inquiry learning effectively in the future.

The empirical studies revealed that IBL can enhance students’ critical thinking skills across various educational levels, from elementary school to higher education students. ^14^ ^–^ ^18^ Although numerous studies have examined the effectiveness of IBL in enhancing critical thinking, the findings remain inconsistent across different contexts, educational levels, and subject areas, teaching strategies, learning media, learning duration, and learning evaluation. ^19^ ^–^ ^21^ Previous meta-analyses have proved that these characteristics act as moderator variables that cause the heterogeneity of effect size of inquiry learning on students’ critical thinking.

However, there are still some gaps. First, previous meta-analyses predominantly focus on STEM disciplines, ^22^ particularly science subjects. ^19^ ^,^ ^23^ Consequently, non-STEM domains such as social sciences, language, culture and arts are underexplored. As a result, the generalizability of findings across broader educational contexts remains unclear.

Second, previous meta-analyses have often overlooked the role of control group variations, even 80% of educational meta-analyses did not include it as a potential moderator. ^24^ In experimental studies, control group conditions vary substantially, such as teacher-centered approaches (i.e., traditional and conventional instruction) or even other student-centered approaches (i.e., problem based learning and STAD). Empirical evidence revealed that the effectiveness of an intervention is inherently relative to the comparison approaches applied. ^25^ Moreover, studies have shown that neglecting variation instructions in control groups may bias effect size estimates and lead to misleading conclusions. ^26^ Despite its importance, this factor has rarely been systematically examined as a moderator in prior meta-analyses.

In addition, prior meta-analyses exhibit methodological limitations related to the analysis and reporting of moderator effects. Some studies provide limited information on the statistical significance of moderators, ^19^ while others report substantial heterogeneity without conducting moderator analyses to explain the observed variation in effect sizes. ^27^ Thus, these limitations may reduce the interpretability and robustness of meta-analytic findings.

These gaps suggest that there is a need for a more comprehensive and methodologically rigorous meta-analysis. Therefore, the present study aims not only to examine the effectiveness of IBL on improving students’ critical thinking, but also to advance previous research by incorporating control group intervention as a key moderator, as well as broaden the subject area of studies. By addressing these limitations, this study hoped to provide more valid and generalizable estimates of the effectiveness of inquiry learning while considering the educational contexts. Specifically, the objectives of the present meta-analysis are to:

1. Estimate the pooled effect size of inquiry-based learning (IBL) on students’ critical thinking skills.
2. Investigate whether the effectiveness of IBL is moderated by study characteristics, including control group variations, country, education level, and subject area.

## Hypothesis

Based on the research objectives above, the hypotheses in this meta-analysis are formulated as follows.

Hypotheses for the first research objective:

H01: Inquiry based learning has no positive and significant effect on students’ critical thinking.

Ha1: Inquiry based learning has a positive and significant effect on students’ critical thinking.

Hypotheses for the second research objective:

H02a: The effectiveness of inquiry based learning on students’ critical thinking is not moderated by control group variations.

H02b: The effectiveness of inquiry based learning on students’ critical thinking is not moderated by subject domain.

H02c: The effectiveness of inquiry based learning on students’ critical thinking is not moderated by education level.

H02d: The effectiveness of inquiry based learning on students’ critical thinking is not moderated by country of study.

Ha2a: The effectiveness of inquiry based learning on students’ critical thinking is moderated by control group variations..

Ha2b: The effectiveness of inquiry based learning on students’ critical thinking is moderated by subject domain.

Ha2c: The effectiveness of inquiry based learning on students’ critical thinking is moderated by education level.

Ha2d: The effectiveness of inquiry based learning on students’ critical thinking is moderated by country of study.

## Method

### Research design

The research approach used in this study was a quantitative method integrated with meta-analysis. This method was chosen because it aligns with the objective of this research, which is to analyze the effect size of Inquiry-Based Learning (IBL) on students’ critical thinking skills. The PRISMA protocol was applied in the literature search to ensure the transparency and credibility of this meta-analysis. ^28^

### Data analysis

Random effects were applied as the model effect size in this meta-analysis. Since all studies included in this meta-analysis employed experimental designs with contrast groups, the effect size can be estimated using Standardized Mean Difference (SMD). SMD is estimated by dividing the difference between the means in the contrast groups by the pooled standard deviation. ^29^ Furthermore, all studies included in this meta-analysis had different scales. Therefore, SMD was applied to equate the different scales or units of all studies included. ^30^ ^,^ ^31^

Among the 57 studies analyzed in this study, ten studies did not report the standard deviation for either the experimental group or the control group. These missing data can be addressed by contacting the writer or checking the attachment of the studies. ^32^ The researcher had emailed them, but there was no response until the data analysis stage. The alternative solution taken was to calculate the pooled standard deviation using the available statistical data in the study such as *t*-test, *z*-value, and Mean Squared Error. ^29^ ^,^ ^33^

In this meta-analysis, the *metafor* package, developed by (Viechtbauer, 2010), in the R program (2025.09.1 + 401) was used to estimate the individual effect size and its variance, as well as the standard deviation error. Whereas, JASP (0.96.0.0) was used to analyze the pooled effect, subgroup, sensitivity and publication bias, and produce the forest and funnel plot. The analysis was conducted using the restricted maximum likelihood (REML) estimator, with the Knapp–Hartung (KNHA) adjustment to enhance the precision of standard errors and confidence intervals. The effect size of the studies is then interpreted based on the criteria presented in Table 1 below.

**Table 1.** Criteria for interpreting Hedges’ g effect sizes, classifying effect magnitudes from ignored to very large.

Effect size ( *g*)	Interpretation
0.00 *g* < 0,20	Ignored
0,20 ≤ *g* < 0.50	Small
0,50 ≤ *g* < 0.80	Moderate
0,80 ≤ *g* < 1.30	Large
1,30 ≤ *g*	Very large

Since Cohen’s *d* tends to provide a biased population effect size for small samples, ^34^ Hedges’ g was employed due to its bias-correction factor. ^29^ Moreover, Hedges’ *g* provides a more accurate estimate of how much the IBL approach affects students’ critical thinking skills compared to the other methods. ^19^

Heterogeneity of the studies was assessed by Cochran’s *Q* statistic. However, it has limited power to detect true heterogeneity when the number of included studies is small and tends to overestimate when the number of studies is large. ^32^ Therefore, the *I* ^2^ statistic is recommended because it is less dependent on the number of studies and offers a clearer and intuitive interpretation of heterogeneity. ^32^ ^–^ ^35^ The tentative categorization values of *I* ^2^ according to Higgins et al. (2003) are 25% (low), 50% (moderate), and 75% (high).

## Literature collection

**Figure 1.**
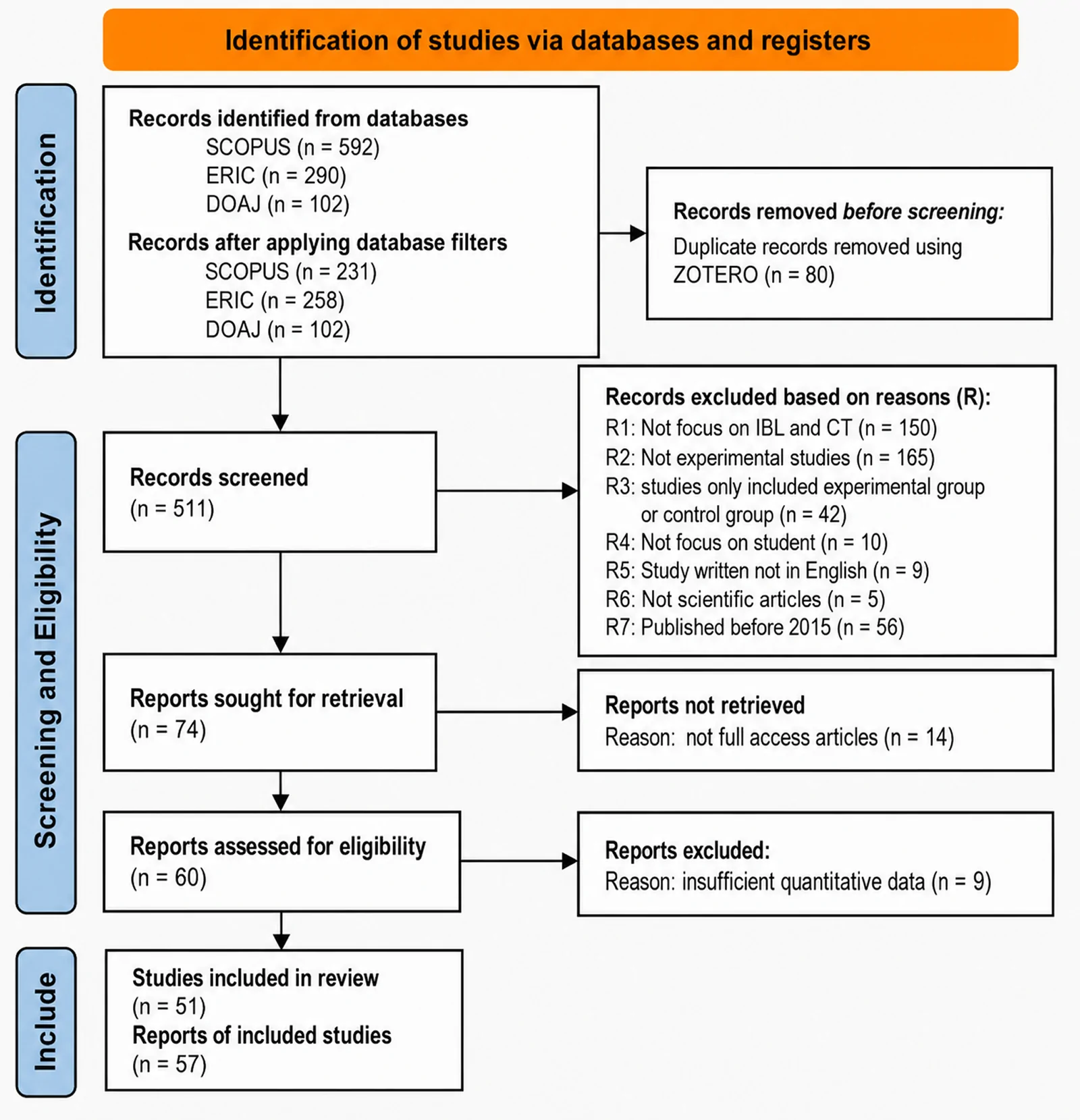
PRISMA flow diagram of the study selection process, showing the number of records identified, screened, excluded, and included, along with reasons (represented by R1-R6) for exclusion at each stage.

The stages of literature collection, which consists of identification, screening and eligibility, and included, are presented in Figure 1.

### Identification

This study began with a systematic collection of relevant literature as data sources through SCOPUS, ERIC, and DOAJ databases. This was done from November 2025 to January 2026.

SCOPUS was selected because it provides credible and high-quality articles. ERIC is one of the most prominent databases for meta-analysis because it provides a wide range of literature in the fields of social and educational sciences, ^36^ ^,^ ^37^ whereas DOAJ was considered because it provides full access to high-quality literature.

The literature search was done using the keywords combined with Boolean Operators (AND, OR) to ensure comprehensive coverage of relevant literature as well as enhance search precision. ^34^ ^,^ ^38^ The literature search was conducted using the following keywords for each database, as shown by Table 2.

**Table 2.** The key words for searching the literature for each database.

Database	Fields	Search strategy	Filter
Scopus	Title, abstract, keywords	((“inquiry-based learning” OR “inquiry based learning” OR “guided inquiry” OR “open inquiry” OR “structured inquiry” OR “inquiry learning” OR “confirmation inquiry”) AND (“critical thinking” OR “critical thinking skills”))	1) Year (2015-2025) 2) Publication stages: final article 3) Source type : journal 4) Language : english 5) Document type: article
ERIC	All fields		1) Publication date: last 20 years 2) Publication type: journal articles 3) Peer review only
DOAJ	All fields	(inquiry-based learning OR inquiry learning OR guided inquiry OR structured inquiry OR open inquiry OR confirmation inquiry) AND effectiveness AND (critical thinking)	Year of publication: 2015–2025

Articles identified from the three databases were screened using database-specific filters. In Scopus, the search was limited to journal articles published within the last ten years, final publication stage, journal article, and English language. In ERIC, the search was limited to peer-reviewed journal articles published within the last 20 years because the database did not provide a “last 10 years” filter option. In DOAJ, the search was limited by publication year within the last ten years. The remaining records are presented in the PRISMA diagram. Metadata from each database were imported into Zotero in RIS format to remove duplicate records and screen article titles and abstracts. Duplicate records may occur because the same article can be indexed in more than one database.

### Screening and eligibility

The remaining articles were screened based on the inclusion and exclusion criteria presented in Table 3.

**Table 3.** Inclusion and exclusion criteria applied in the study selection process.

Inclusion	Exclusion
True/quasi-experimental design	Pre-experimental or qualitative design
Focus on IBL and CT	Not focused on IBL and CT
Participants are from elementary to higher education students	Non-student populations or outside specified levels
Sufficient quantitative data	Insufficient quantitative data
Published between 2015–2025	Published before 2015

Based on Figure 1, ten studies were excluded because their subjects were focused on teachers. Although the Scopus search was restricted to English-language articles, nine Indonesian-language articles were identified from other databases, particularly DOAJ, which indexes articles in various languages.

A total of 74 studies proceeded to the full-text retrieval stage. At this stage, only open-access and downloadable articles were considered. The downloadable articles were read thoroughly to determine the sufficient amount of substantial data. The eligible articles were then extracted into the coding table.

### Included

A total of 51 independent studies were included, yielding 57 study entries. The number of study entries exceeded the number of independent studies because Lue et al (2020) ^39^ reported four statistical datasets, whereas Prayogi et al (2024), ^40^ Dewi et al (2021), ^41^ and Duran & Dokme (2016) ^42^ each reported two effect sizes.

### Data coding and risk of bias assessment

**Data coding**

The substantial data, extracted from post-test data of both experimental and control groups of each study, ^43^ were tabulated into the coding table, which consists of the number of samples, mean, and standard deviation of experimental and control groups. ^21^ ^,^ ^36^ In addition, moderator variables such as control group variations, subject area, country and education level were also coded. ^29^ The coding procedures were conducted by two coders to ensure their accuracy and reliability. ^44^

The first coder (AH) and the second coder (TSK) independently exctracted all of included studies. The discrepancies were resolved through discussion until consensus was reached. Cohen’s kappa was used to assess inter-coder reliability because the coded variables in this meta-analysis were categorical. ^45^

**Risk of bias assessment (RoB)**

Risk of bias was assessed independently by two reviewers for all included studies using the revised JBI Critical Appraisal Checklist for Quasi-Experimental Studies developed by Baker et al (2024). ^46^ This tool was selected because most of the included studies employed quasi-experimental designs. This tool evaluates internal validity (eight items) and statistical conclusion validity (one item). Based on these domains, each study was classified as having a low, moderate, or high risk of bias. Since Baker et al (2024) did not specify explicit criteria for these overall risk categories, we used RoB criteria by Krasny-Pacini et al (2014) ^47^ as presented by Table 4. For each included study, each risk-of-bias (RoB) item was rated as “Yes” or “No” and coded as 1 or 0, respectively. An internal validity score was calculated by dividing the sum of all item scores by the total number of RoB items. The resulting score was interpreted according to the criteria presented in Table 4.

**Table 4.** Risk-of-Bias criteria developed by Krasny-Pacini et al. (2014).

Criteria	Interpretation
>75%	Low risk of bias
>50–75%	Moderate
≤50%	High risk of bias

Cohen’s kappa was calculated from the initial independent ratings of the two reviewers before consensus discussion.

## Findings and discussion

### Coding data and Risk of bias assessment (RoB)

**Coding data**

Inter-rater reliability was assessed using Cohen’s kappa for each categorical coding variable. According to the criteria proposed by Landis & Koch (1977), ^45^ the results indicated almost perfect agreement between the two coders for subject domain, κ = 0.855, country, κ = 0.856, education level, κ = 0.881, and control group variatons, κ = 0.861. The overall Cohen’s kappa was 0.896, also indicating almost perfect agreement. These findings suggest that the coding procedure was consistent between coders before disagreements were resolved through discussion.

**Risk of bias assessment (RoB)**

Based on Landis & Koch (1977)‘s criteria, ^45^ the resulting Cohen’s kappa coefficient of 0.73 is classified as substantial agreement between the reviewers. Overall, most included studies were classified as low risk. Of the 51 studies assessed, 46 were rated as low risk and 5 were rated as moderate risk. No study was classified as high risk. The studies rated as moderate risk were Maharani et al (2023), ^48^ Subagiyo et al (2023), ^17^ Chususiyah et al (2020), ^49^ Nurza et al (2021), ^50^ and Wiwik et al (2025). ^51^ At the effect-size level, 52 of the 57 effect sizes were derived from low-risk studies, while 5 were derived from moderate-risk studies. Overall, these findings indicate that most included studies had adequate methodological quality for meta-analysis, as the majority were classified as low risk and no study was rated as high risk.

### Descriptive

The characteristic of each study is presented in Table 5. Overall, the studies involved a total of 3.628 students consisting of 907 elementary school students, 545 junior high school students, 1.205 senior high school students, and 971 higher education students. Based on the group, there were 1.818 students in the experimental group and 1.810 students in the control group.

**Table 5.** Study characteristics. Summary of included studies and moderator variables. Some studies –Lu et al (2020), Prayogi et al (2024), Dewi et al (2021), Duran & Dokme (2016) – reported more than one effect sizes, indicated by suffixes (a–d).

Study	N	g	SE	Subject Domains	Country	Education level	Control group variations
Wulandari (2022)	160	1,55	0,18	NSL	Indonesia	SHS	TCL
Rahmi et al (2019)	64	1,04	0,27	NSL	Indonesia	JHS	TCL
Gunawan et al (2019)	64	1,48	1,06	NSL	Indonesia	JHS	TCL
Astina et al (2025)	36	2,17	0,42	SSL	Indonesia	SHS	TCL
Mbhanyisi et al (2025)	46	1,21	0,32	NSL	South Africa	SHS	TCL
Maharani et al (2023)	53	2,19	0,35	NSL	Indonesia	ES	SCL
Latifah & Suprihatiningrum (2024)	72	0,75	0,24	NSL	Indonesia	SHS	SCL
Ghaemi & Mirsaeed (2017)	40	1,94	0,38	LL	Iran	HE	TCL
Arsal (2017)	56	0,23	0,27	SSL	Turkey	HE	TCL
Farah & Ayoubi (2020)	38	2,81	0,46	NSL	Lebanon	JHS	TCL
Lu et al (2020a)	53	0,54	0,28	NSL	Lebanon	SHS	TCL
Lu et al (2020b)	53	1,04	0,29	NSL	Taiwan	ES	TCL
Lu et al (2020c)	58	1,1	0,28	NSL	Taiwan	JHS	TCL
Lu et al (2020d)	58	1,64	0,3	NSL	Taiwan	ES	TCL
Subagiyo et al (2023)	65	1,9	0,3	NSL	Indonesia	SHS	TCL
Syafaren et al (2019)	80	1,42	0,25	NSL	Indonesia	JHS	TCL
Mayarni et al (2023)	54	2,73	0,38	NSL	Indonesia	ES	TCL
Styawan & Arty (2020)	50	0,75	0,29	NSL	Indonesia	SHS	SCL
Lestari & Anggraini (2021)	60	1,97	0,31	LL	Indonesia	JHS	TCL
Chususiyah et al (2020)	42	5,18	0,64	LL	Indonesia	JHS	TCL
Azizah & Umah (2025)	36	2,83	0,47	NSL	Indonesia	ES	TCL
Khasawneh et al (2022)	41	1,23	0,34	Math	America	HE	TCL
Yue et al (2023)	57	0,62	0,27	LL	China	HE	SCL
Carracedo (2025)	54	0,28	0,27	LL	Spain	HE	TCL
Pahrudin et al (2021)	50	1,2	0,31	NSL	Indonesia	SHS	SCL
Gombo (2025)	60	1,72	0,3	Math	Indonesia	SHS	TCL
Ritli & Adlini (2022)	52	1,62	0,32	NSL	Indonesia	SHS	TCL
Sholikhan & Kusnadi (2021)	128	1,27	0,19	NSL	Indonesia	SHS	TCL
Damayanti (2025)	72	3,12	0,35	SSL	Indonesia	SHS	TCL
Sucilestari & Arizona	60	2,22	0,33	NSL	Indonesia	HE	SCL
Musyawwir et al (2023)	56	1,08	0,29	NSL	Indonesia	ES	TCL
Purwanita et al (2019)	50	1,32	0,31	SSL	Indonesia	ES	TCL
Aido et al (2022)	100	0,81	0,21	NSL	Ghana	HE	TCL
Nurhalisa & Rahmawaty (2025)	68	0,94	0,26	NSL	Indonesia	JHS	TCL
Irwanto et al (2018)	48	2,22	0,37	NSL	Indonesia	HE	TCL
Irwanto (2023)	64	1,72	0,29	NSL	Indonesia	HE	TCL
Ramirez (2021)	71	0,58	0,24	NSL	Philippines	JHS	TCL
Prayogi et al (2024a)	54	0,34	0,27	NSL	Indonesia	HE	TCL
Prayogi et al (2024b)	55	1,22	0,29	NSL	Indonesia	HE	TCL
Mitarlis et al (2020)	66	1,3	0,27	NSL	Indonesia	HE	TCL
Zhou et al (2025)	55	1,41	0,3	Math	China	ES	TCL
Dewi et al (2021a)	50	0,71	0,29	NSL	Indonesia	HE	SCL
Dewi et al (2021b)	76	0,87	0,24	NSL	Indonesia	HE	SCL
Hasan et al (2019)	65	2,42	0,33	NSL	Indonesia	SHS	SCL
Ismail et al (2025)	53	0,49	0,28	NSL	Indonesia	ES	SCL
Hussein et al (2019)	127	1,14	0,19	NSL	Malaysia	ES	TCL
Pattipeilohy et al (2022)	102	0,89	0,21	NSL	Indonesia	SHS	SCL
Duran and Dökme (2016a)	47	1,53	0,33	NSL	Turkey	ES	TCL
Duran and Dökme (2016b)	43	1,59	0,35	NSL	Turkey	ES	TCL
Nurza et al (2021)	56	1,38	0,3	NSL	Indonesia	ES	TCL
Poerwanti et al (2022)	108	−2,27	0,25	SSL	Indonesia	ES	SCL
Putra et al (2018)	64	−0,61	0,26	NSL	Indonesia	SHS	SCL
Mitarlis et al (2020)	66	4,22	0,44	NSL	Indonesia	HE	TCL
Doyan et al (2023)	62	0,85	0,27	NSL	Indonesia	SHS	TCL
Aiman et al (2020)	58	0,88	0,28	NSL	Indonesia	ES	TCL
Wiwik et al (2025)	68	1,03	0,26	NSL	Indonesia	SHS	SCL
Djatmiko et al (2020)	84	0,6	0,22	NSL	Indonesia	HE	SCL

*Note*: N: total sample, *g*: Hedge’s g effect size, SE: standard error, ES:elementary school, JHS: junior high school, SHS: senior high school, HE: higher education, NSL: Natural Science Learning, SSL: Social Science Learning, Math: Mathematics, LL: Language Learning, SCL: Student-Centered Learning, TCL: Teacher-Centered Learning.

### Summary effect size

**Figure 2.**
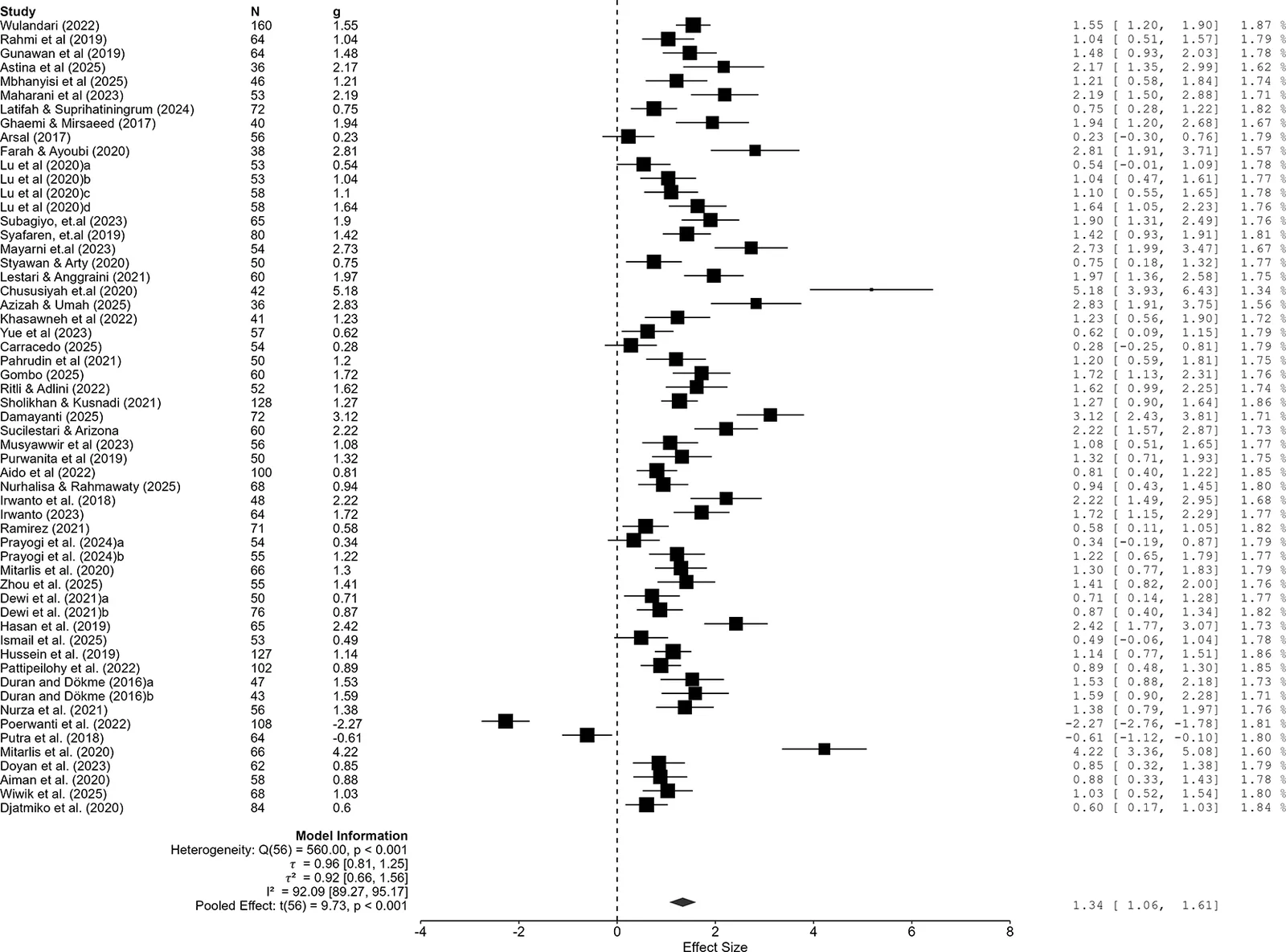
Forest Plot. Forest plot of Hedges’ g for 57 effect sizes derived from 51 studies. Squares represent individual effect sizes (proportional to study weight), horizontal lines indicate 95% confidence intervals, and the diamond represents the pooled effect size.

Figure 2 represents the forest plot that encompasses the substantial statistics data analyzed in this meta-analysis, such as individual effect size along with its study weight and confidence interval (CI), summary effect size, and statistics heterogeneity. It is shown that the summary effect size estimated using a random-effects model is 1,336 with a *p*-value <0,001. Based on Hedge’s *g* criteria, this value indicates that IBL has a large and significant effect on students’ critical thinking (CT).

The forest plot also demonstrates that all studies included in this meta-analysis have positive and statistically significant pooled effects, as indicated by confidence intervals (CI) 95% ranged from 1.061 to 1.611 that do not cross the line of zero. ^19^ ^,^ ^52^ However, study by Putra et al (2018), ^53^ Poerwanti et al (2022), ^54^ Ismail & Isroktum (2025), ^55^ Farah et al (2020), ^56^ Carracedo (2025), ^57^ Arsal (2017) ^14^ and Lu et al. (2020a) ^39^ show insignificant effect, as indicated by the CI that crosses the zero line.

The summary effect size is based on weighted individual effect sizes of the whole studies, with effect sizes from larger samples weighted more than the effect sizes from smaller samples. ^52^ A study by Wulandari et al. (2022) ^58^ has the largest contribution to the summary effect size, indicated by the highest study weight of 1.87%. Conversely, the study by Farah and Ayoubi (2020) ^56^ gives the lowest contribution indicated by the lowest study weight (1.57%).

The heterogeneity analysis indicated substantial variability among the included studies. ^19^ ^,^ ^34^ This was shown by Cochran’s *Q* and I ^2^ tests. Cochran’s *Q* test shows statistically significant heterogeneity *Q* (56) = 560.00, p < 0.001. It means that the observed variability in effect sizes cannot be explained solely by sampling error. ^30^ Similarly, the high value of the I ^2^ statistic shows that 92.09% of the total variability in effect sizes was caused by real differences between studies rather than random variation. Collectively, these statistics consistently indicate substantial heterogeneity and the subsequent exploration of potential moderator variables. ^59^

The findings of this meta-analysis show positive and significant summary effects of the thirty-seven studies regarding the effect of inquiry-based learning on students’ critical thinking skills, with a Hedge’s g = 1.34. Based on Cohen’s d criteria, this effect is classified as a very large effect. It means that students who engage in IBL demonstrate substantially higher critical thinking skills compared to those receiving traditional or other learning models applied in the fifty seven studies. This finding confirms that IBL can be a potential learning model in enhancing student’s critical thinking skills.

This result is larger than that of the previous meta-analysis by Arifin et al. (2025) ^19^ (g (N = 36) = 1.27; 95% CI [0.78, 1.76]). Despite showing large and significant effects, their meta-analysis was limited only on studies conducted on natural science subjects. In contrast, our meta-analysis covered the studies in the field of natural science, mathematics, social science, and language. Therefore, with the large summary effect size, it can be inferred that IBL is not only effective in natural science learning, but also in mathematics, social science and language. Nevertheless, this meta-analysis confirms the meta-analysis by Arifin et al. (2025) ^19^ in terms of summary effect and confidence interval. It is shown that the summary effect size of this meta-analysis is still in the range of confidence interval of pooled effect size of their meta-analysis. Therefore, the present meta-analysis is consistent with the previous meta-analysis.

The strong effect of IBL on critical thinking can be explained through its core pedagogical characteristics. Unlike traditional methods, which often emphasize the transmission of factual knowledge, inquiry based learning engages students in some activities enhancing students critical thinking, such as constructing knowledge, explaining, reasoning, questioning, and communicating with their instructor or their peers. ^60^ These activities are the substantial component of critical thinking. Overall, the results suggest that teachers should apply IBL in the classroom in order to enhance students’ critical thinking.

Despite a large and significant pooled effect, the individual studies included in this meta-analysis showed considerable variation in effect sizes. For instance, a study by Arsal (2017) ^14^ shows negligible effect (g = 0.2321; 95% CI [−0.29,0.76]). In contrast, study by Chususiyah et al (2020) shows a very large effect (g = 5.18; 95% CI [3.93, 6.43]).

This finding indicates that the effectiveness of IBL on critical thinking may depend on some factors acting as the moderator variables. Thus, this meta-analysis not only analyse the overall effect size, but also detected the potential moderator variables that are responsible for the variety of effect size across the studies, including control group variations, level of education, subject, and country.

### Moderator analysis

Moderator analysis was conducted to examine the sources of the heterogeneity of effect size, ^52^ ^,^ ^61^ that may influence the effectiveness of IBL on critical thinking in this meta-analysis. Since the moderator variables considered in this meta-analysis are categorical moderators, the subgroup analysis was applied to analyze moderator variables. ^62^ Recent meta-analysis complements the methodological limitation of the previous meta-analysis by reporting the significance of the subgroup analysis. The subgroups were divided into four groups, i.e., control group variations, subject area, level of education, and country.

### Control group variations

The studies included in this meta-analysis compared inquiry-based learning (IBL) with several types of control-group instruction, such as traditional, conventional, expository, cooperative learning, problem-based learning (PBL), project-based learning (PjBL), and communicative language teaching (CLT). Because traditional and conventional instruction are overlap conceptually, we grouped the control-group approaches into two broader categories, that is, teacher-centered learning (TCL) and student-centered learning (SCL). Based on, ^63^ traditional, conventional, and expository instruction were categorized as TCL because these approaches generally place the teacher as the main source of explanation and as the primary controller of learning content, activities, materials, and pacing. Similarly, expository instruction was also included in the TCL category because it is closely related to direct instructional guidance, where teachers explicitly present the concepts and procedures that students are expected to learn. ^64^

In contrast, cooperative learning, PBL, PjBL, and CLT were categorized as SCL because they provide more space for students to participate actively in the learning process. According to Goodwin, these approaches typically involve collaboration, discussion, exploration, authentic problem solving, communication, and learner engagement. In the same way, CLT engages students as communicators, negotiators, and contributors through activities such as pair/group work, discussion, role play, and problem solving. ^65^ IBL itself is classified as an SCL approach. Therefore, in this subgroup analysis, we examined whether the effect of IBL differed depending on whether it was compared with other SCL instructions or with TCL instructions.

The subgroup analysis showed that the type of control-group learning approach significantly moderated the effect of IBL on students’ critical thinking, Qm (1) = 5.21, p = .022. Studies comparing IBL with TCL produced a larger effect size, k = 42; g = 1.527, than studies comparing IBL with SCL, k = 15; g = 0.779.

These findings suggest that the effectiveness of IBL depends partly on the instructional approach used in the control group. IBL tends to demonstrate a greater effect when compared with teacher-centered approaches. This result aligns with the meta-analysis by Arifin et al. (2025), which found that IBL had a large effect on improving students’ critical thinking skills compared with conventional instruction. In contrast, the effect of IBL was relatively smaller when compared with other student-centered approaches. This result is supported by Wijnia et al (2024), ^66^ who found that student-centered, problem-driven approaches such as PBL, PjBL, and CBL had positive effects compared with teacher-centered instruction, but did not differ significantly from one another. Therefore, the smaller effect of IBL when compared with other SCL approaches may be explained by the fact that IBL and other student-centered approaches share several core characteristics, such as active participation, collaboration, problem solving, and knowledge construction.

### Country of study

The studies included in this meta-analysis were conducted across twelve countries. However, seven countries, namely South Africa, Iran, the United States, Malaysia, Ghana, the Philippines, and Spain, were represented by only one study each. To ensure sufficient statistical stability and to prevent the exclusion of these studies from the analysis, they were combined into a single “Other countries” subgroup. The subgroup analysis revealed no statistically significant differences between country subgroups, Qm (5) = 3.33, p = .650, indicating that the effectiveness of inquiry-based learning (IBL) on students’ critical thinking was relatively consistent across national contexts.

All country subgroups showed positive pooled effects of IBL on students’ critical thinking. The largest pooled effect was found in studies conducted in Lebanon, k = 2; g = 1.646; 95% CI [−12.77, 16.06]. Yet, the extremely wide and included zero confidence interval indicates substantial statistical imprecision. Indonesia also showed a large and statistically significant positive effect, k = 40; g = 1.429; 95% CI [1.054, 1.804], followed by Taiwan, k = 3; g = 1.250; 95% CI [0.442, 2.058]. Turkey showed a positive but imprecise effect, k = 3; g = 1.096, 95% CI [−0.834, 3.025], while China also showed a positive estimate, k = 2; g = 1.004; 95% CI [−4.013, 6.021]. Yet, with a wide confidence interval crossing zero indicates substantial statistical imprecision. The “Other countries” subgroup showed the smallest but still positive and statistically significant pooled effect, k = 7; g = 0.983; 95% CI [0.512, 1.453]. Thus, although the magnitude of the pooled estimates varied across countries, the direction of effect was consistently positive.

This finding is consistent with study by Santhosh et al (2023), ^67^ who found that study location did not significantly moderate the effectiveness of informal STEM project-based learning. Although Santhosh et al (2023) focused on PjBL rather than IBL, their finding remains relevant because both approaches are student-centered and involve active investigation, problem solving, collaboration, and reflection. These processes are closely related to critical thinking. Thus, the finding provides indirect support that the effectiveness of student-centered learning may depend more on fidelity of implementation than on geographical context. ^68^

However, this result should be interpreted cautiously because several country subgroups contained few studies and the number of studies across countries was unbalanced. According to Cuijpres et al (2021), ^61^ subgroup analyses with small or unequal numbers of studies often have limited statistical power to detect reliable moderator effects.

### Subject domains

The subject domains represented in the included studies varied considerably. While some studies reported broad subject domains, others focused on specific subjects, including physics, biology, chemistry, economics, history, mathematics, and psychology. Because psychology, history, and social science were each represented by only one study, retaining them as separate categories would have resulted in their exclusion from the subgroup analysis in JASP. Therefore, all subject areas were grouped into four broader subject domains, that is, natural sciences, social sciences, mathematics, and language learning to ensure adequate subgroup representation and facilitate meaningful comparisons.

The subgroup analysis showed no significant differences in the effect of IBL across subject domains, Qm (3) = 1.43; p = .698. This indicates that subject domains did not significantly moderate the effect of IBL on students’ critical thinking. One possible explanation for this result is that IBL is not designed for a specific subject domain, but can be adapted across different subject areas. This finding is consistent with meta-analysis by Antonio & Prudente (2024), ^44^ which found that scientific discipline did not significantly moderate the effect of inquiry-based learning on students’ higher-order thinking skills in science learning. Based on the subgroup effect size, the largest effect was found in language learning, k = 5; g = 1.939; 95% CI [−0.415, 4.294], followed by mathematics, k = 5; g = 1.471; 95% CI [0.863, 2.079], natural science learning, k = 44; g = 1.313; 95% CI [1.072, 1.553], and social science, k = 5; g = 0.901; 95% CI [−1.679, 3.481]. However, the pattern of confidence intervals suggests an important distinction. The effects in natural science learning and mathematics were more precisely estimated, whereas the estimates for language learning and social science were much less precise, as indicated by wide confidence intervals that crossed zero.

One possible explanation for this insignificant result is that IBL is not designed for a specific subject domain, but can be adapted across different subject areas. Kuhlthau (2010) ^69^ described inquiry as a way of learning that is increasingly used across all subject areas of the curriculum and emphasized that Guided Inquiry can support students’ learning of curriculum content across subject domains. Similarly, Pedaste et al (2015) ^70^ described IBL as a structured inquiry cycle consisting of orientation, conceptualization, investigation, conclusion, and discussion. Thus, when these inquiry processes are implemented appropriately, IBL may support learning across subject domains.

### Education levels

The subjects of the studies analyzed in this meta-analysis were distributed into four levels of education – from elementary school to higher education. The subgroup analysis indicated that IBL produced positive effects on students’ critical thinking across all education levels. The largest effect was observed at the junior high school level (k = 9, g = 1.757, 95% CI [0.728, 2.785]), followed by senior high school (k = 17, g = 1.296, 95% CI [0.868, 1.724]), higher education (k = 16, g = 1.250, 95% CI [0.721, 1.779]), and elementary school (k = 15, g = 1.244, 95% CI [0.595, 1.893]). However, the test of subgroup differences was not significant, Qm (3) = 1.11, p = .774, indicating that education grade did not significantly moderate the effect of IBL on students’ critical thinking.

A possible explanation is that IBL effectiveness may depend more on fidelity of implementation than on students’ education level. A prior meta-analysis by Furtak et al (2012) ^71^ emphasized that IBL should be understood in terms of the cognitive features of the learning activity and the degree of guidance provided to students. Their meta-analysis also showed that teacher-led inquiry produced stronger effects than student-led inquiry, suggesting that structured guidance is central to effective IBL. Similarly, Öztürk et al (2022) ^72^ found that grade level did not significantly moderate the effect of IBL on learning outcomes, while Antonio & Prudente (2024) ^44^ reported that IBL positively affected students’ higher-order thinking skills regardless of educational level. Thus, although the point estimates varied across education levels, these differences were not statistically meaningful, suggesting that education grade itself did not systematically explain variation in IBL effectiveness.

The substantial heterogeneity observed in this meta-analysis may be partly explained by several study-level characteristics that were not formally tested as moderators, including implementation fidelity, type of inquiry, intervention duration, and assessment instruments. Previous meta-analysis evidence supports the plausibility of these factors. For example, Antonio & Prudente (2024) ^44^ found that the effectiveness of inquiry-based approaches varied across inquiry levels, with open inquiry showing the largest effect size, followed by guided and structured inquiry, although the subgroup difference was not statistically significant. Lazonder & Harmsen (2016) ^68^ further showed that the type of guidance can moderate performance success in inquiry learning, indicating that the level and form of instructional support may influence learning outcomes.

Intervention duration may also contribute to heterogeneity. Antonio & Prudente (2024) ^44^ reported significant subgroup differences by implementation duration, although the pattern was not strictly linear. This suggests that longer interventions do not automatically produce stronger effects; rather, duration may interact with instructional intensity, student engagement, and implementation quality. Similarly, Lazonder & Harmsen (2016) ^68^ treated duration as a moderator by distinguishing single-session from multiple-session inquiry interventions.

Implementation fidelity is another plausible source of effect sizes heterogeneity. Although fidelity was not commonly coded as a formal moderator in previous IBL meta-analyses, related evidence indicates that the quality of instructional support matters. Lazonder & Harmsen (2016) ^68^ found positive effects of guidance in inquiry learning, while Alfieri et al (2010) ^73^ showed that enhanced discovery approaches involving feedback, scaffolding, worked examples, and elicited explanations were more effective than unassisted discovery. Therefore, differences in teachers’ adherence to inquiry procedures, scaffolding practices, questioning strategies, and support for evidence-based reasoning may partly explain variability in effect sizes.

Finally, assessment instruments may influence the magnitude of the observed effects. Arifin et al (2025) ^19^ included evaluation as a subgroup variable in a meta-analysis of IBL and critical thinking, while broader meta-analyses on critical thinking have treated instruments or measuring tools as potential moderators. Researcher-developed or curriculum-aligned assessments may be more sensitive to the intervention content and therefore yield larger effects, whereas standardized critical-thinking instruments may provide more conservative estimates. Taken together, these findings suggest that the remaining heterogeneity in the present meta-analysis should be interpreted as reflecting meaningful differences in how inquiry-based learning was implemented, structured, sustained, and assessed across studies.

### Sensitivity analysis

Sensitivity analysis was conducted using standardized residual diagnostics and leave-one-out influence diagnostics to assess whether the pooled effect was robust to outlying and influential studies. Following Viechtbauer & Cheung (2010), ^74^ outliers and influential cases should be examined in meta-analysis because their presence may affect the validity and robustness of meta-analytic conclusions. The studies by Chususiyah et al (2020), ^49^ Poerwanti et al (2022), ^54^ and Mitarlis et al (2020) ^75^ were identified as potential outliers because their standardized residuals exceeded the absolute value of 3, and they were also identified as influential cases in the diagnostic output. Chususiyah et al. (2020), Poerwanti et al. (2022), and Mitarlis et al. (2020) had standardized residuals of 3.527, −4.081, and 3.697, respectively. Before exclusion, the pooled effect was significant, g = 1.336, 95% CI [1.061, 1.611], with very high heterogeneity, I ^2^ = 92.09%. After excluding the three studies, the pooled effect slightly decreased but remained significant, g = 1.285, 95% CI [1.089, 1.480], while heterogeneity decreased to I ^2^ = 83.87%. These findings indicate that the main conclusion was robust to the influence of the identified studies, although substantial heterogeneity remained.

### Publication bias

**Figure 3.**
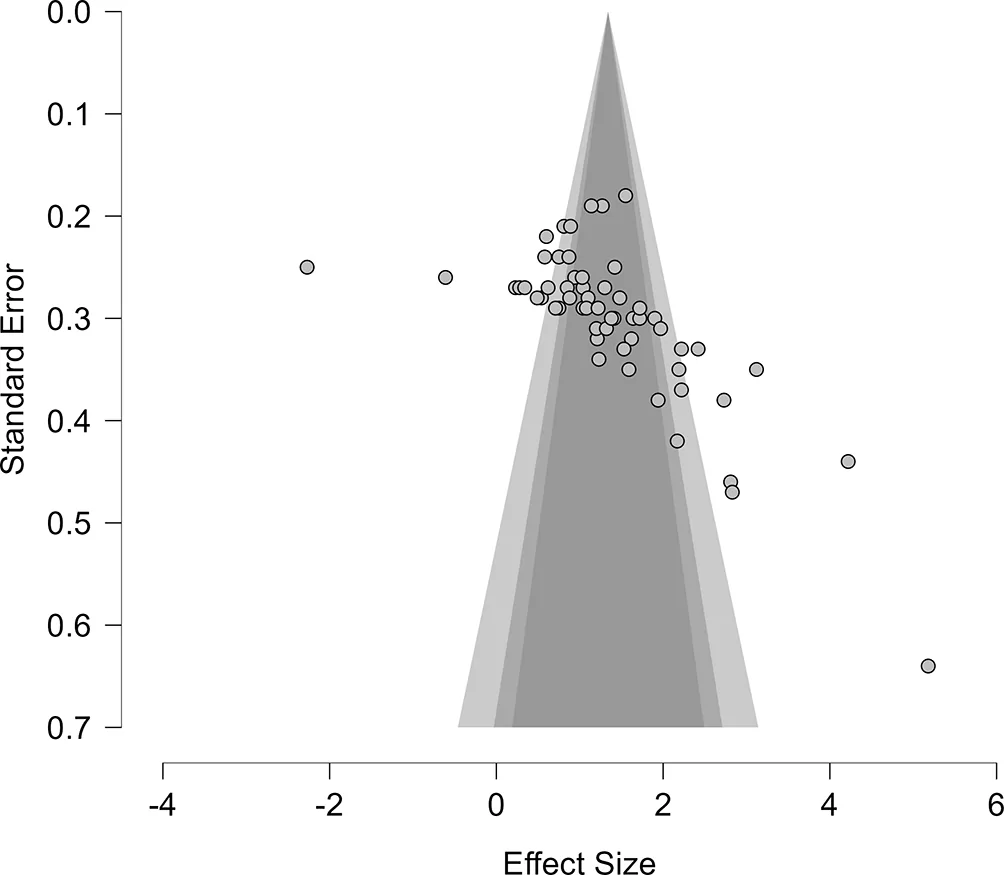
Funnel plot of standardized mean differences (Hedges’ g) for the included studies. The distribution of effect sizes is asymmetrical, suggesting potential small study effects and the possibility of publication bias.

In the absence of publication bias or small study effects, the estimated individual effect sizes tend to distribute symmetrically on both sides around the pooled effect size. ^29^ The funnel plot in Figure 3 demonstrates the asymmetrical plot, as indicated by the small studies that tend to distribute on the right side. This pattern suggests potential small-study effects and possibility of publication bias. However, since the interpretation of funnel plot tends to be subjective, ^76^ Egger’s Regression test is needed as a further analysis to quantify the asymmetry of the funnel plot. ^29^

The publication bias analysis showed that all of the methods applied to assess the publication bias were statistically significant, as shown by Table 6 below.

**Table 6.** Results of publication bias assessment, including Fail-Safe N, Kendall’s Tau, and Egger’s regression test.

Test name	Value	p
Fail-Safe N	22013	-
Kendall’s Tau	0.557	<.001
Egger’s Regression	−1.003	<.001

Based on Table 6, it is shown that Fail-Safe N shows a very large number, meaning that an extremely high number of unpublished studies with null findings would be required to nullify the observed effect. This suggests that this meta-analysis is statistically robust and not easily overturned by potential missing evidence. ^77^ However, robustness alone does not guarantee the absence of publication bias. Kendall’s Tau suggests a systematic association between effect sizes and their variances. ^78^ In practical terms, this means that studies with lower precision (typically smaller studies) tend to report larger effects. ^79^ ^,^ ^80^ This pattern is widely recognized as small-study effects, which are often linked to selective publication or reporting practices in meta-analysis. ^29^

The asymmetrical funnel plot is confirmed by the Egger’s regression test. A significant result in Egger’s test indicates asymmetry in the funnel plot, suggesting that smaller studies yield systematically larger effect sizes than expected under a symmetric distribution. ^81^

**Figure 4.**
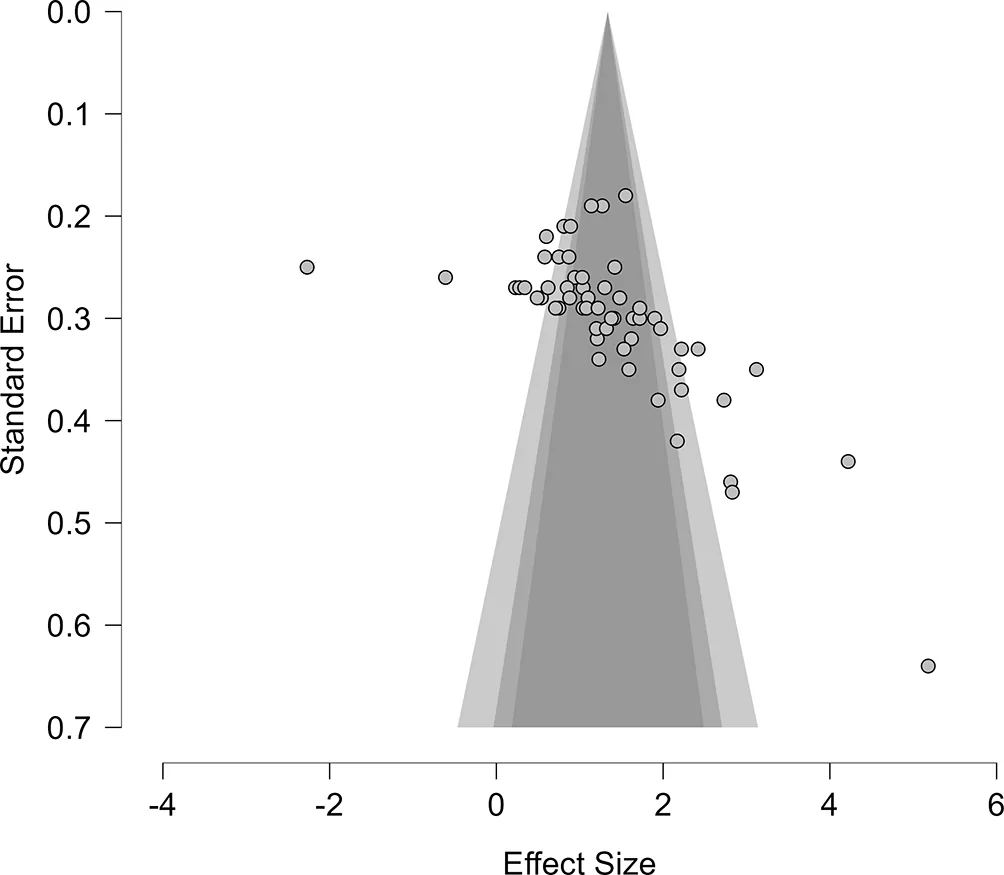
Funnel plot after applying the Trim and Fill procedure. No studies were imputed, and the pooled effect size remained unchanged, supporting the possibility of publication bias.

Since the Egger’s test was statistically significant, the analysis was processed to trim and fill the test to identify the missing studies. ^76^ The trim and fill test showed that there were no missing studies found in this meta-analysis, as shown visually in Figure 4. After the trim and fill process, the pooled effect size does not change (g = 1.336; p < .0001; 95% CI [1.074, 1.597]). Moreover, the heterogeneity remains the same (i.e., I ^2^ = 92.09%). Figure 3 and Figure 4 above demonstrate the funnel plot before and after the trim and fill. It is shown clearly that the funnel plot is still the same, which shows visually that there were no missing studies in this meta-analysis.

The asymmetrical funnel plot shows that there is a possibility of publication bias in this meta-analysis. The Egger’s Regression test confirms statistically the presence of publication bias shown by the funnel plot. Yet, Trim and Fill analysis showed no missing studies. Funnel plot asymmetry and a significant Egger’s test indicate the presence of small-study effects but do not constitute definitive evidence of publication bias. ^82^ Such asymmetry may also arise from between-study heterogeneity, methodological differences, or contextual variations. Moreover, given the limited sensitivity of the trim-and-fill method under heterogeneous conditions, the absence of imputed studies suggests that the observed asymmetry is more likely attributable to study characteristics rather than publication bias alone. ^83^

### Results of hypothesis testing

Regarding the effect of IBL on students’ critical thinking, it is shown that IBL has a large and significant effect on students’ critical thinking. Therefore, for the first research objective the null hypothesis (H01) was rejected and the alternative hypothesis (Ha1) was accepted. Whereas, for the second research objective, the moderator testing showed that only control group variations significantly moderated the effectiveness of IBL on students’ critical thinking. Thus, the null hypothesis (H02a) was rejected and the alternative hypothesis (Ha2a) was accepted. In contrast, subject domain, education level, and country of study did not significantly moderate the effectiveness of IBL on students’ critical thinking. Therefore, the null hypotheses (H02b, H02c, and H02d) were accepted and the alternative hypotheses (Ha2b, Ha2c, and Ha2d) were rejected.

## Conclusion

The present meta-analysis demonstrates that Inquiry-Based Learning (IBL) has a large and statistically significant positive effect on students’ critical thinking skills, indicating that students engaged in IBL generally achieve higher critical thinking outcomes than those in comparison groups. Subgroup analysis showed that the type of control group variations significantly moderated the effect of IBL, meaning that its effect was relative to the comparison group. In other words, IBL has a larger effect when compared with teacher-centered learning than when compared with other student-centered learning. This suggests that IBL’s advantage is more pronounced when the comparison condition relies mainly on teacher explanation, direct instruction, or conventional classroom practices, whereas its relative effect is smaller when compared with approaches that share similar student-centered features, such as active participation, collaboration, problem solving, and knowledge construction. In contrast, country, subject domain, and education level did not significantly moderate the effect of IBL. Although effect sizes varied descriptively across these subgroups, the differences were not statistically significant. Thus, the findings should not be interpreted as evidence that IBL is more effective in a particular country, subject domain, or education level. Rather, IBL appears to have broadly positive effects across diverse contexts, although this conclusion should be interpreted cautiously because some subgroup estimates were based on small or uneven numbers of studies.

Publication bias assessment indicated funnel plot asymmetry and potential small-study effects, as shown by the funnel plot and significant Kendall’s Tau and Egger’s regression test. However, these results do not provide definitive evidence of publication bias, as funnel plot asymmetry may also reflect heterogeneity, methodological differences, or contextual variation. The trim-and-fill analysis did not impute missing studies or change the pooled effect estimate, suggesting that the overall effect was not clearly driven by missing-study bias, although publication bias cannot be completely ruled out. Sensitivity analysis further supported the robustness of the main finding. After excluding three outlying and influential studies, the pooled effect decreased slightly but remained large, positive, and statistically significant. Heterogeneity also decreased but remained substantial, indicating that the excluded studies contributed to between-study variability without altering the overall conclusion. These findings support IBL as an effective instructional approach for enhancing students’ critical thinking skills. Its advantage appears strongest when compared with teacher-centered instruction, while its effectiveness is generally consistent across countries, subject domains, and education levels. Nevertheless, the findings should be interpreted with caution because substantial heterogeneity and potential small-study effects remained.

### Future directions

Future research is recommended to examine additional potential moderators that may explain the remaining heterogeneity in the effect of IBL on students’ critical thinking skills, particularly implementation fidelity, type or level of inquiry, intervention duration, and assessment instruments. These variables are important because IBL may produce different effects depending on how consistently inquiry procedures are implemented, how much guidance or autonomy is provided to students, how long the intervention is delivered, and how critical thinking is measured.

## Data availability

### Underlying data

Zenodo. Does Inquiry-Based Learning Improve Students’ Critical Thinking? A Meta-Analysis Accounting for Control Group Variations [Data set]. https://doi.org/10.5281/zenodo.21246615. ^84^

The project contains the following underlying data:

• dataset.xlsx (List of all studies with relevant details for statistical analysis)
• Risk of Bias assessment.xlsx

## Extended data

This project contains the following extended data:

• Figure 1 – PRISMA Flowchart.tif
• Figure 2 – Forest plot.tiff
• Figure 3 – Funnel plot trim and fill.tiff
• Figure 4 – Funnel plot.tiff
• PRISMA 2020 – checklist abstract and review.docx
• PRISMA 2020 – abstract checklist.docx
• Script for computating effect size
• R script Risk of Bias Assessment
• R script reliability of coding data

All data are available under the terms of the Creative Commons Attribution 4.0 International (CC-BY 4.0) license.
