# Supplementary material for: Does Inquiry-Based Learning Improve Students’ Critical Thinking? A Meta-Analysis Accounting for Control Group Variations
Source: F1000Res. 2026 Sep 8;15:730. Originally published 2026 May 14. [Version 3] doi: 10.12688/f1000research.180569.3 (PMC13562932; doi:10.12688/f1000research.180569.3)
Supplement: Peer review history

# Reviewer report: Reviewer response for version 3

**DOI:** 10.5256/f1000research.209542.r519172

**Published:** 2026-09-10

**Authors:** Torang Siregar

**Affiliations:** 1 Department of Mathematics Education, Faculty of Tarbiyah and Teacher Training (FTIK), UIN Syekh Ali Hasan Ahmad Addary Padangsidimpuan, Padangsidimpuan, North Sumatra, Indonesia; 2 Department of Mathematics Education, Padang State University (UNP), Padang, West Sumatra, Indonesia

I have carefully reviewed the revised version of the manuscript and the authors’ responses to the reviewers’ comments. I am satisfied that the concerns raised in my previous review have been adequately addressed. The revisions have improved the clarity, methodological transparency, and overall quality of the manuscript.

 I have no further substantive comments or revisions to request. I therefore support the indexing of the revised manuscript.

Are the rationale for, and objectives of, the Systematic Review clearly stated?

Yes

Is the statistical analysis and its interpretation appropriate?

Yes

If this is a Living Systematic Review, is the ‘living’ method appropriate and is the search schedule clearly defined and justified? (‘Living Systematic Review’ or a variation of this term should be included in the title.)

Not applicable

Are sufficient details of the methods and analysis provided to allow replication by others?

Partly

Are the conclusions drawn adequately supported by the results presented in the review?

Partly

Reviewer Expertise:

My Area(s) of Research:Educational Research and EvaluationMathematics EducationMeta-Analysis and Systematic Review MethodologyCritical Thinking and Higher-Order Thinking SkillsInquiry-Based and Student-Centered LearningQuantitative Research Methods in Education

I confirm that I have read this submission and believe that I have an appropriate level of expertise to confirm that it is of an acceptable scientific standard.

# Reviewer report: Reviewer response for version 2

**DOI:** 10.5256/f1000research.205847.r509110

**Published:** 2026-08-20

**Authors:** Konstantinos T. Kotsis

**Affiliations:** 1 University of Ioannina, Ioannina, Greece

The authors have responded substantially and constructively to the comments raised in my initial review. The revised manuscript has been considerably strengthened, particularly in relation to the search strategy, study coding, risk-of-bias assessment, and interpretation of the statistical findings.

 The expanded search identified additional eligible studies, increasing the evidence base to 57 study entries from 51 independent articles. The authors have also added a formal risk-of-bias assessment using the JBI Critical Appraisal Checklist for Quasi-Experimental Studies. The coding process is now described more transparently, with two independent coders and the reporting of inter-rater reliability using Cohen’s kappa. Furthermore, the classification of the comparison conditions has been improved by grouping overlapping “traditional” and “conventional” approaches into the broader categories of teacher-centred and student-centred learning.

 The statistical analyses have been revised appropriately. The updated pooled effect size and moderator analyses are reported more accurately, and the authors now acknowledge the substantial heterogeneity and possible small-study effects. Importantly, the revised manuscript no longer presents subject domain, educational level, and country as significant moderators. The conclusions are consequently more closely aligned with the reported results and appropriately emphasize the importance of the comparison condition.

 Nevertheless, a few issues still require attention before the manuscript can be fully approved:

1. The discussion should address more explicitly the possible sources of the substantial residual heterogeneity, including the type and level of inquiry, implementation fidelity, intervention duration, teacher guidance, and the instruments used to assess critical thinking.
2. The risk-of-bias findings should be connected more clearly to the interpretation of the pooled effect. The authors should explain whether studies with higher risk of bias influenced the magnitude or robustness of the overall estimate.

Overall, the authors have adequately addressed the major methodological concerns raised in the first review. The remaining issues are limited and can be resolved through a focused final revision.

 Recommendation: Approved with Reservations.

Are the rationale for, and objectives of, the Systematic Review clearly stated?

Yes

Is the statistical analysis and its interpretation appropriate?

Partly

If this is a Living Systematic Review, is the ‘living’ method appropriate and is the search schedule clearly defined and justified? (‘Living Systematic Review’ or a variation of this term should be included in the title.)

Not applicable

Are sufficient details of the methods and analysis provided to allow replication by others?

Partly

Are the conclusions drawn adequately supported by the results presented in the review?

Partly

Reviewer Expertise:

My research focuses on physics education and science education, with particular interest in inquiry-based learning, students’ misconceptions, scientific literacy, preservice teacher education, artificial intelligence in science teaching, and the use of real-world contexts to support conceptual understanding in physics and science.

I confirm that I have read this submission and believe that I have an appropriate level of expertise to confirm that it is of an acceptable scientific standard, however I have significant reservations, as outlined above.

# Response

**Published:** 2026-08-28

**Authors:** Abel Harapan

Thank you for your constructive comments. We have revised our article in response to your comments.

1. Regarding the possible sources of residual heterogeneity, we have addressed them explicitly, item by item. However, we did not discuss “teacher guidance” separately because we consider teacher guidance to be an integral part of, and therefore covered by, the level of inquiry.
2. Regarding the risk of bias, we conducted an additional sensitivity analysis to examine the impact of studies with a moderate risk of bias on the robustness of the pooled effect.

# Reviewer report: Reviewer response for version 2

**DOI:** 10.5256/f1000research.205847.r510241

**Published:** 2026-08-13

**Authors:** Torang Siregar

**Affiliations:** 1 Department of Mathematics Education, Faculty of Tarbiyah and Teacher Training (FTIK), UIN Syekh Ali Hasan Ahmad Addary Padangsidimpuan, Padangsidimpuan, North Sumatra, Indonesia; 2 Department of Mathematics Education, Padang State University (UNP), Padang, West Sumatra, Indonesia

**1. Summary of the Article**

 This meta-analysis investigates the effectiveness of Inquiry-Based Learning (IBL) in improving students' critical thinking skills. The authors synthesized 57 effect sizes from 51 studies published between 2015–2025, using a random-effects model with Hedges' *g* as the effect size metric. The study extends previous meta-analyses by examining control group variations as a potential moderator, alongside country, education level, and subject domain. The analysis revealed a large and significant pooled effect (*g* = 1.336), with substantial heterogeneity ( *I²* = 92.09%). Moderator analysis showed that only control group variation significantly moderated the effect, with IBL showing larger effects when compared to teacher-centered learning (*g* = 1.527) than when compared to other student-centered approaches (*g* = 0.779). Country, education level, and subject domain were not significant moderators. The authors also conducted risk-of-bias assessment, sensitivity analysis, and publication bias diagnostics.

**2. Detailed Reviewer Comments**

***2.1. Rationale and Objectives (Answer: Yes)***

**Strengths:**

- The rationale is clearly articulated. The authors effectively position IBL within the broader context of critical thinking development in the digital age.
- The research gap is precisely identified: previous meta-analyses have (a) focused predominantly on STEM/science subjects, (b) overlooked control group variations as a moderator, and (c) exhibited methodological limitations in reporting moderator effects.
- The objectives are explicitly stated and directly aligned with the identified gaps.
- Hypotheses are clearly formulated in both null and alternative forms, enhancing transparency.

**No revisions needed on this aspect.**

***2.2. Methods and Analysis (Answer: Partly)***

**Strengths:**

- The study follows the PRISMA 2020 protocol, which strengthens methodological transparency.
- Inclusion/exclusion criteria are clearly presented in a dedicated table.
- The use of Hedges' *g*, random-effects model, REML estimation, and Knapp-Hartung adjustment are appropriate statistical choices.
- The authors conducted a formal risk-of-bias assessment using the revised JBI Critical Appraisal Checklist, with inter-rater agreement reported (Cohen's κ = 0.73 for RoB, and 0.855–0.896 for coding variables).
- Sensitivity analysis and publication bias diagnostics (Fail-Safe N, Kendall's Tau, Egger's test, Trim-and-Fill) were performed, which strengthens the robustness of the findings.
- The authors provide open data and R scripts, supporting reproducibility.

**Limitations Requiring Attention:**

1. **Search Strategy Completeness (Critical):**
  1. Although the authors expanded the search strings in Version 2, the search strategy still lacks **complete, database-specific search strings**. The manuscript reports keywords (Table 2) but does not provide the exact Boolean search strings used for Scopus, ERIC, and DOAJ.
  2. **Recommendation:** Provide the full search strings for each database in an appendix or supplementary material, including field restrictions (e.g., TITLE-ABS-KEY for Scopus), filters applied, and the exact dates of the searches.
2. **Search Date:**
  1. The exact date(s) on which the literature searches were conducted are not reported.
  2. **Recommendation:** State the specific date(s) of the search (e.g., "The literature search was conducted on [DD Month YYYY]").
3. **Coding Procedure Detail:**
  1. While the authors now report inter-rater reliability using Cohen's kappa, the manuscript does not fully explain **how coding disagreements were resolved** beyond stating "through discussion until consensus was reached."
  2. **Recommendation:** Provide a brief description of the consensus process, including whether a third coder was consulted if disagreements persisted.
4. **Risk of Bias Classification Criteria:**
  1. The authors used criteria from Krasny-Pacini et al. (2014) to classify studies as low, moderate, or high risk of bias. However, Baker et al. (2024) did not specify explicit criteria for overall risk categories.
  2. **Recommendation:** Justify why the Krasny-Pacini criteria were chosen and acknowledge that this is an adaptation rather than a standard part of the JBI tool.

***2.3. Statistical Analysis and Interpretation (Answer: Yes)***

**Strengths:**

- The statistical procedures are appropriate and well-executed.
- Heterogeneity is formally assessed using both Cochran's *Q* and *I²*, with the substantial heterogeneity ( *I²* = 92.09%) appropriately acknowledged.
- The moderator analysis is conducted systematically, with *Qm* statistics and *p*-values reported for each moderator.
- The authors correctly interpret that the effect of IBL is relative to the comparison group – a nuanced and important finding.
- Publication bias is assessed using multiple complementary methods, and the authors provide a balanced interpretation, noting that funnel plot asymmetry may also reflect heterogeneity and methodological differences rather than definitive publication bias.

**Minor Suggestions:**

1. **Interpretation of Subgroup with Few Studies:**
  1. The authors acknowledge that some subgroups (e.g., Lebanon, *k* = 2; Other countries, *k* = 7) have small numbers of studies. However, the interpretation could be more explicit that findings from these subgroups are **exploratory** rather than confirmatory.
  2. **Recommendation:** Add a sentence stating that subgroup analyses with fewer than 10 studies should be interpreted with caution and considered hypothesis-generating.
2. **Very Large Effect Size:**
  1. The pooled effect (*g* = 1.336) is very large. The authors appropriately note heterogeneity, but they could also discuss whether this effect size is **plausible** in the context of educational interventions, where effect sizes above 0.80 are relatively rare.
  2. **Recommendation:** Briefly comment on the plausibility of the large effect size, considering that researcher-developed assessments (which may be more sensitive to the intervention) were likely used in many included studies.

***2.4. Conclusions (Answer: Partly)***

**Strengths:**

- The main conclusion – that IBL has a positive and significant effect on critical thinking – is supported by the pooled effect size.
- The conclusion that the effect of IBL depends on the comparison group (larger vs. teacher-centered, smaller vs. student-centered) is well-grounded in the subgroup analysis.
- The authors appropriately acknowledge that country, subject domain, and education level did not significantly moderate the effect, and they do not overstate these findings.

**Areas Requiring Revision:**

1. **Overgeneralization of IBL Effectiveness:**
  1. While the conclusion states that IBL "has a large and statistically significant positive effect," the substantial heterogeneity ( *I²* = 92.09%) suggests that this average effect may not apply uniformly across all contexts.
  2. **Recommendation:** Revise the conclusion to emphasize that IBL is **on average** effective, but its effectiveness varies considerably depending on implementation, comparison group, and study conditions. For example:

"On average, IBL demonstrates a large positive effect on students' critical thinking skills. However, the substantial heterogeneity observed indicates that the effectiveness of IBL varies considerably across contexts, and the pooled estimate should be interpreted as a general trend rather than a universal effect."

1. **Practical Implications:**
  1. The conclusions are somewhat limited to statistical findings and do not offer clear practical implications for educators or policymakers.
  2. **Recommendation:** Add a brief paragraph on practical implications, such as:
    - IBL is most effective when compared to teacher-centered instruction, suggesting that adopting IBL can significantly enhance critical thinking in contexts where traditional instruction predominates.
    - The smaller effect compared to other student-centered approaches suggests that the pedagogical approach itself may be less important than the **active, student-centered features** shared across these methods.
2. **Limitations Should Be More Explicit:**
  1. The authors discuss heterogeneity and publication bias, but the limitations section could be strengthened by explicitly stating:
    - The majority of studies were conducted in Indonesia (*k* = 40), which may limit generalizability.
    - The lack of standardized critical thinking assessments across studies may influence effect size estimates.
  2. **Recommendation:** Add these points to the Conclusion or a separate Limitations subsection.

**3. Specific Points for Revision (Summary)**

**Issue**

**Recommendation**

 Incomplete search strings

 Provide complete, database-specific search strings in an appendix or supplementary material.

 Search date not reported

 Report the exact date(s) when the literature search was conducted.

 Coding disagreement resolution

 Briefly describe the consensus process (e.g., whether a third coder was involved).

 RoB classification criteria justification

 Justify the use of Krasny-Pacini criteria and acknowledge it as an adaptation.

 Overgeneralized conclusion

 Revise the conclusion to emphasize that the effect is **on average** and varies across contexts.

 Practical implications absent

 Add practical implications for educators and policymakers.

 Limitations understated

 Explicitly state limitations related to geographic concentration and assessment heterogeneity.

 Small subgroup interpretation

 Clarify that findings from subgroups with few studies (e.g., Lebanon, Other countries) are exploratory.

***Recommendation***

 This is a well-conducted meta-analysis that makes a valuable contribution to the literature on IBL and critical thinking. The authors have substantially improved the manuscript in Version 2 by adding risk-of-bias assessment, sensitivity analysis, inter-rater reliability, and expanding the search strategy. However, several methodological details (particularly complete search strings and coding procedures) need to be clarified to ensure full reproducibility. The conclusions should also be tempered to reflect the substantial heterogeneity and the exploratory nature of some subgroup analyses.

**Recommendation:** **Minor Revision**

***By. Reviewer***

Are the rationale for, and objectives of, the Systematic Review clearly stated?

Yes

Is the statistical analysis and its interpretation appropriate?

Yes

If this is a Living Systematic Review, is the ‘living’ method appropriate and is the search schedule clearly defined and justified? (‘Living Systematic Review’ or a variation of this term should be included in the title.)

Not applicable

Are sufficient details of the methods and analysis provided to allow replication by others?

Partly

Are the conclusions drawn adequately supported by the results presented in the review?

Partly

Reviewer Expertise:

My Area(s) of Research:Educational Research and EvaluationMathematics EducationMeta-Analysis and Systematic Review MethodologyCritical Thinking and Higher-Order Thinking SkillsInquiry-Based and Student-Centered LearningQuantitative Research Methods in Education

I confirm that I have read this submission and believe that I have an appropriate level of expertise to confirm that it is of an acceptable scientific standard, however I have significant reservations, as outlined above.

# Response

**Published:** 2026-08-28

**Authors:** Abel Harapan

Thank you for your constructive comments. We have revised our article in response to your comments.

1. Regarding the search date and time, we have added the overall search period for the literature search, which was conducted from 30 November 2025 to 14 January 2026. We did not report separate search dates for each database because the searches were conducted iteratively across the databases within this overall period rather than on fixed, pre-specified dates for each database. We believe that reporting the overall search period provides an accurate representation of the search process. In addition, the search strings and filters applied to each database have been explicitly reported in Table 2 to facilitate reproducibility.
2. Regarding the practical implications, we have revised the article by adding a discussion of the practical implications of our findings. We have focused these implications specifically on teachers because they are the primary actors responsible for implementing inquiry-based learning (IBL) in classroom practice.

# Reviewer report: Reviewer response for version 1

**DOI:** 10.5256/f1000research.199188.r488709

**Published:** 2026-06-05

**Authors:** Baiduri Baiduri

**Affiliations:** 1 Mathematics Education, Universitas Muhammadiyah Malang Fakultas Ilmu Kesehatan, Malang, East Java, Indonesia

**Reviewer Report**

**Baiduri Baiduri**

**Univeristas Muhammadiyah Malang, Indonesia**

**1. Are the rationale for, and objectives of, the systematic review clearly stated?**

 The rationale for the review is clearly articulated. The manuscript highlights the growing importance of critical thinking within educational contexts and positions Inquiry-Based Learning (IBL) as a pedagogical approach with the potential to foster higher-order thinking skills (p. 3). This discussion provides a convincing basis for the review and situates the study within ongoing scholarly conversations on critical thinking and inquiry-oriented instruction.

 The authors also define the research gap with sufficient clarity. They note that previous meta-analyses have largely concentrated on STEM and science education, have paid limited attention to variation in control-group conditions as a potential moderator, and have been constrained by methodological issues related to moderator analyses and the reporting of heterogeneity (p. 3). These observations establish a clear need for further investigation and provide a coherent justification for the present study.

 The objectives of the review are explicitly stated and closely aligned with the identified gaps. Specifically, the study seeks to estimate the overall effect of Inquiry-Based Learning on students’ critical thinking and to determine whether this effect differs according to control-group variation, country, educational level, and subject area (p. 3). The progression from the literature review to the research objectives is logical and well developed.

 The hypotheses are presented clearly and correspond directly to the stated aims of the study (p. 4). Together, they offer a coherent structure for the analyses that follow and enhance the overall transparency of the research design.

**Strengths**

 A notable strength of the manuscript lies in the way the rationale is developed and anchored in the existing literature. The research gap is identified with precision and is convincingly connected to the objectives of the review. The objectives themselves are specific and sufficiently focused to address the limitations highlighted in earlier studies. The hypotheses are likewise clearly formulated and remain consistent with the analytical framework adopted throughout the manuscript.

**Limitations**

 No substantive concerns were identified regarding the rationale, research objectives, or hypotheses based on the information provided in the manuscript.

**Assessment:** **Yes**

**2. Are sufficient details of the methods and analysis provided to allow replication by others?**

 The methodological approach is described in a generally clear and systematic manner. The study is framed as a meta-analysis conducted in accordance with PRISMA guidelines, which provides an established structure for the identification, screening, and selection of relevant studies (pp. 4–5). The literature search drew on three databases: Scopus, ERIC, and DOAJ (p. 5) and employed the keywords “inquiry-based learning” AND “student critical thinking” (p. 6).

 The inclusion and exclusion criteria are reported in a dedicated table, making the eligibility requirements transparent and easy to follow (p. 6). The study-selection process is also documented through a PRISMA flow diagram, enabling readers to trace the progression from the initial pool of records to the final set of studies included in the analysis (p. 5).

 The manuscript outlines the coding framework and identifies the variables incorporated into the analysis, including the moderator variables examined in the study (p. 6). Statistical analyses were conducted using the *metafor* package in R and JASP (p. 4). The authors describe the procedures used to estimate effect sizes, including the calculation of Hedges’ *g*, the application of Restricted Maximum Likelihood (REML) estimation, and the use of the Knapp–Hartung adjustment (p. 4). The treatment of studies with missing standard deviations is also explained, with alternative statistical information from the original studies used when necessary (p. 4).

**Strengths**

 The methodological procedures are presented in a reasonably transparent and organized manner. The eligibility criteria and study-selection process are clearly documented, allowing readers to understand how studies were identified and screened. Key analytical decisions, including effect-size estimation procedures, statistical models, and software packages, are explicitly reported. The inclusion of a PRISMA flow diagram further strengthens transparency by providing a concise overview of the selection process and the derivation of the final sample.

**Limitations**

 Despite the generally adequate methodological description, several details that would support full reproducibility are either absent or only briefly addressed. Although the keywords used in the search are reported, the manuscript does not provide the complete search strings applied across the individual databases (p. 6). The dates on which the searches were conducted are likewise omitted (pp. 5–6), making it difficult to determine the temporal coverage of the review.

 The coding procedure also receives limited attention. While the manuscript indicates that coding was undertaken by the first author under the supervision of the second author, it offers little information about how coding decisions were implemented or verified (p. 6). In particular, no evidence is provided regarding coding agreement, inter-coder reliability, or procedures used to resolve potential discrepancies. The absence of these details limits the extent to which the coding process can be independently replicated.

**Assessment:** **Partly**

 The manuscript provides a clear account of the main methodological and analytical procedures and offers sufficient information to understand how the meta-analysis was conducted. However, important details related to the search strategy and coding process are not fully reported, which restricts the reproducibility of the review.

**3. Is the statistical analysis and its interpretation appropriate?**

 The statistical procedures employed in this meta-analysis are generally appropriate and align well with the study’s objectives. The authors used a random-effects model to estimate the overall effect size, a suitable choice given the likelihood of variability across educational contexts, participant characteristics, and implementation conditions among the included studies (p. 4). The use of Hedges’ *g* as the effect-size metric is also appropriate, particularly for synthesizing findings from experimental and quasi-experimental research with differing sample sizes (p. 4).

 The manuscript reports formal assessments of heterogeneity using both Cochran’s *Q* statistic and the *I²* index (pp. 4, 8), allowing readers to evaluate the extent to which variation among study outcomes exceeds what would be expected by sampling error alone. To investigate potential sources of this variability, the authors conducted moderator analyses based on control-group variation, country, educational level, and subject area. Relevant *Qm* statistics and significance values are reported, enabling readers to assess the contribution of each moderator to between-study differences (pp. 10–11).

 The evaluation of publication bias is similarly thorough. Rather than relying on a single diagnostic approach, the authors employed multiple complementary procedures, including funnel-plot inspection, Fail-Safe N analysis, Kendall’s Tau, Egger’s regression test, and Trim-and-Fill analysis (pp. 11–12). This combination of methods provides a more robust basis for assessing the potential influence of publication bias on the overall results.

**Strengths**

 The analytical framework is consistent with established meta-analytic practice and is appropriately matched to the research questions. Key statistical procedures are clearly identified and sufficiently described. Heterogeneity is formally assessed, moderator analyses are conducted in a systematic manner, and publication bias is examined through multiple complementary techniques. Taken together, these elements contribute to the methodological rigor of the study and strengthen confidence in the analytical process.

**Limitations**

 Although the statistical procedures themselves are appropriate, several aspects of the results warrant a more cautious interpretation. The reported heterogeneity is substantial, with an *I²* value of 83.84% (p. 8), indicating considerable variability among the included studies. Such a high level of heterogeneity suggests that the pooled effect size should be interpreted as an average across diverse contexts rather than as a uniformly applicable estimate.

 The explanatory power of the moderator analyses is also somewhat limited by the available evidence base. Several moderator categories were excluded because they contained too few studies to support reliable subgroup comparisons (pp. 10–11). In particular, the country-level analysis was restricted to studies conducted in Indonesia and Taiwan, as studies from other countries did not meet the minimum subgroup requirements (p. 10). This limitation reduces the extent to which conclusions regarding geographical differences can be generalized.

 In addition, the publication-bias analyses yielded mixed evidence. While some indicators suggest robustness, funnel-plot asymmetry was observed, and both Kendall’s Tau and Egger’s regression test produced statistically significant results (pp. 11–12). These findings indicate the possibility of publication bias and should therefore be considered when interpreting the magnitude of the reported effects.

**Assessment:** **Partly**

 The statistical analyses are appropriate, clearly reported, and generally interpreted in a manner consistent with the study objectives. However, the substantial heterogeneity observed across studies, the restricted scope of several moderator analyses, and indications of potential publication bias introduce important limitations that should be acknowledged when drawing conclusions from the findings.

**4. Are the conclusions drawn adequately supported by the results presented in the review?**

 The conclusions presented in the manuscript are generally well supported by the findings of the meta-analysis. The authors conclude that Inquiry-Based Learning (IBL) has a positive effect on students’ critical thinking, and this interpretation is consistent with the reported pooled effect size of *g* = 1.48 and the associated 95% confidence interval of [1.23, 1.73], which indicates a statistically significant positive effect (pp. 8–9, 13). The conclusion follows directly from the quantitative evidence and is appropriately grounded in the reported results.

 The manuscript further argues that the effectiveness of IBL is influenced by contextual factors, including control-group variation, country, educational level, and subject area. These claims are based on the subgroup analyses presented in the results section (pp. 10–11) and are linked to the corresponding statistical evidence. The discussion does not appear to introduce conclusions that extend beyond the analyses that were conducted.

 Another positive aspect is that the authors acknowledge the complexity of the evidence base rather than focusing exclusively on the overall effect size. The discussion recognizes the substantial heterogeneity observed across studies and incorporates this issue into the interpretation of the findings (pp. 8–13). The results of the publication-bias assessment are likewise reported and considered when evaluating the strength of the evidence (pp. 11–13). This contributes to a more balanced interpretation of the results.

**Strengths**

 The principal conclusions are closely aligned with the findings reported in the meta-analysis. Claims regarding the overall effectiveness of IBL are directly supported by the pooled effect-size estimates, while conclusions concerning moderator effects are derived from the subgroup analyses presented in the results section. The discussion situates these findings within a broader educational context without substantially departing from the available evidence. The manuscript also demonstrates an awareness of methodological considerations by acknowledging heterogeneity and discussing the results of the publication-bias analyses.

**Limitations**

 Although the conclusions are generally supported by the reported findings, some moderator-related interpretations should be viewed with caution. The country-level analysis is based only on studies conducted in Indonesia and Taiwan (pp. 10, 13), which limits the extent to which conclusions about geographical variation can be generalized. Consequently, the observed differences may reflect characteristics of the available evidence rather than broader international patterns.

 A further limitation concerns the uneven distribution of studies across moderator categories. Several categories did not contain a sufficient number of studies to permit subgroup analysis and were therefore excluded from the investigation (pp. 10–11). As a result, the moderator findings represent only a subset of the potentially relevant contextual factors influencing the effectiveness of IBL. These constraints should be more explicitly acknowledged when interpreting the scope and generalizability of the conclusions.

**Assessment:** **Partly**

 Overall, the conclusions are adequately supported by the results presented in the review and remain largely consistent with the statistical evidence. Nevertheless, some conclusions related to moderator effects are derived from a limited evidence base and should therefore be interpreted within the constraints of the available studies and subgroup distributions.

**Recommendation:** **Major Revision**

**Main Reasons**

1. Complete database-specific search strings are not reported.
2. The dates of the literature search are not reported.
3. Additional details regarding the coding procedure are not provided.
4. Coding agreement or reliability measures are not reported.
5. The study reports substantial heterogeneity ( *I²* = 83.84%).
6. Several moderator categories contained insufficient studies for subgroup analysis.
7. The publication-bias analyses reported funnel-plot asymmetry together with statistically significant Kendall’s Tau and Egger’s regression results.
8. A Conflict of Interest (Competing Interests) statement is not reported in the manuscript.

**Overall Comment**

 This manuscript addresses a meaningful question in educational research by examining the extent to which Inquiry-Based Learning contributes to the development of students’ critical thinking skills. The study is grounded in a clearly articulated rationale, and its objectives are well aligned with the gaps identified in previous meta-analytic work. One notable contribution is the inclusion of control-group variation as a moderator, alongside country, educational level, and subject area, which broadens the scope of existing evidence. The review procedures and analytical approach are generally described in a clear and transparent manner, and the statistical methods are appropriate for the questions being investigated.

 At the same time, several aspects of the methodological reporting would benefit from greater detail. The manuscript reports the search keywords used in the literature search but does not provide the full search strings for each database, nor does it specify the dates on which the searches were conducted. Information regarding the coding process is also limited, particularly with respect to coding procedures and evidence of coding reliability. The results indicate substantial heterogeneity among the included studies, and several moderator analyses were constrained by the small number of studies available within particular categories. In addition, the publication-bias assessment identified funnel-plot asymmetry and statistically significant results for both Kendall’s Tau and Egger’s regression.

 Providing more comprehensive information on these methodological and reporting aspects would improve the transparency and reproducibility of the review and allow readers to assess the strength of the evidence with greater confidence.

Are the rationale for, and objectives of, the Systematic Review clearly stated?

Yes

Is the statistical analysis and its interpretation appropriate?

Partly

If this is a Living Systematic Review, is the ‘living’ method appropriate and is the search schedule clearly defined and justified? (‘Living Systematic Review’ or a variation of this term should be included in the title.)

Not applicable

Are sufficient details of the methods and analysis provided to allow replication by others?

Partly

Are the conclusions drawn adequately supported by the results presented in the review?

Partly

Reviewer Expertise:

Teaching and learning, cognitive psychology, problem solving, thinking processes, especially in mathematics education

I confirm that I have read this submission and believe that I have an appropriate level of expertise to confirm that it is of an acceptable scientific standard, however I have significant reservations, as outlined above.

# Response

**Published:** 2026-07-10

**Authors:** Abel Harapan

We sincerely thank for your constructive comments and valuable suggestions. Your feedback has helped us improve the quality, transparency, and rigor of our manuscript. The revisions made in response to your comments are summarized below.

 1. Search strategy

 We expanded the search strings for each database and presented them in a table, including the applied filters, field restrictions, and search dates. As a result, 51 independent studies were included, yielding 57 effect sizes because several studies reported more than one effect size. Accordingly, we re-ran the meta-analysis and revised the results, moderator analyses, and interpretation.

 2. Coding procedure and agreement

 We added information explaining that the coding process was conducted by two independent coders. Any discrepancies were resolved through discussion. Inter-rater reliability was assessed using Cohen’s kappa

 3. Sufficient studies for subgroup analysis

 Regarding the insufficient number of studies in some subgroups, we attempted to identify additional studies by expanding the search string. As a result, 57 reports were included, which provided a sufficient number of studies for several subgroup analyses, such as type of control group variation, educational level, and subject domain. However, for the country-of-study subgroup, seven countries—South Africa, Iran, the United States, Malaysia, Ghana, the Philippines, and Spain—were each represented by only one study. To improve statistical stability and avoid excluding these studies from the analysis, we combined them into a single “Other countries” subgroup.

 4. Publication bias

 We revised the interpretation of publication bias to make it more cautious and balanced. In addition, we conducted and reported a sensitivity analysis to further assess the robustness of the meta-analysis findings. We have added a “Competing Interests” section at the end of the manuscript.

 We also updated the extended materials by adding evidence of inter-rater coding reliability, the risk-of-bias assessment, the sensitivity analysis, and the R script used to calculate Cohen’s kappa. In addition, we clarified the coding procedures and revised the interpretation of heterogeneity, publication bias, small-study effects, and moderator analyses. We hope these revisions have improved the transparency, reproducibility, and methodological rigor of the manuscript.

# Reviewer report: Reviewer response for version 1

**DOI:** 10.5256/f1000research.199188.r485795

**Published:** 2026-05-28

**Authors:** Konstantinos T. Kotsis

**Affiliations:** 1 University of Ioannina, Ioannina, Greece

The manuscript addresses an important and timely issue in education research: whether inquiry-based learning improves students’ critical thinking skills and whether the type of control group used in primary studies affects the estimated intervention effect. The topic is relevant, especially because inquiry-based learning is widely promoted as a student-centred pedagogical approach, but its effectiveness often varies across subjects, educational levels, and instructional contexts.

 This manuscript is a **systematic review with meta-analysis**. It follows the general structure of a systematic review, uses the PRISMA 2020 framework, reports inclusion and exclusion criteria, presents a PRISMA flow diagram, and synthesizes quantitative findings using meta-analytic procedures. The use of Hedges’ g, a random-effects model, heterogeneity statistics, moderator analysis, forest plot, funnel plot, and publication-bias diagnostics are appropriate methodological choices for a study of this nature.

**Explaining the Responses to the Journal's questions for Reviewers**

***Are the rationale for, and objectives of, the Systematic Review clearly stated?***

**Yes.** The rationale and objectives are clearly stated. The authors explain that previous meta-analyses have mainly focused on STEM or science subjects and have not adequately considered variation in control-group conditions as a moderator. They therefore justify the need for a broader meta-analysis examining the effect of inquiry-based learning on students’ critical thinking and the moderating role of control-group variation, country, education level, and subject area. The two objectives are explicitly listed at the end of the Introduction.

***Are sufficient details of the methods and analysis provided to allow replication by others?***

**Partly.** The manuscript provides useful methodological details, including the databases searched, the use of PRISMA, inclusion and exclusion criteria, coding categories, effect-size metric, random-effects model, REML estimation, Knapp–Hartung adjustment, and publication-bias tests. However, replication would be difficult in its current form because the search strategy is too limited and not fully reproducible. The authors report using the keywords **“inquiry-based learning” AND “student critical thinking”**, but they do not provide complete search strings for each database, exact search dates, field restrictions, filters, or a sufficiently detailed screening protocol. The coding process also needs more detail, especially regarding independent coders, disagreement resolution, and intercoder reliability. Therefore, the methods are reasonably described but not yet sufficiently detailed for full replication.

***Is the statistical analysis and its interpretation appropriate?***

**Partly.** The use of Hedges’ g, a random-effects model, heterogeneity statistics, moderator analysis, and publication-bias diagnostics is generally appropriate for a meta-analysis of intervention studies. However, the interpretation requires more caution. The pooled effect size is very large, **g = 1.48**, while heterogeneity is also substantial, **I² = 83.84%**, suggesting that the studies differ considerably. In addition, the funnel plot and Egger’s regression indicate asymmetry or small-study effects, which may affect the reliability of the pooled estimate. Some subgroup analyses are based on small numbers of studies, and several categories were excluded because of insufficient data. For this reason, the statistical procedures are mostly appropriate, but the interpretation is sometimes too strong and should be more cautious.

***Are the conclusions drawn adequately supported by the results presented in the review?***

**Partly.** The conclusion that inquiry-based learning has a positive effect on students’ critical thinking is broadly supported by the reported pooled effect. The moderator findings also support the idea that effectiveness varies according to control group, subject area, education level, and country. However, some conclusions are stated too generally. Because heterogeneity is high, publication-bias indicators are significant, and some moderator subgroups are small or unstable, the authors should avoid strong claims that inquiry-based learning is uniformly powerful across contexts. The conclusions should be revised to emphasize that IBL appears promising, but its effectiveness depends on implementation conditions, comparison group, educational level, subject domain, and the methodological quality of the included studies.

**Reviewer Final Evaluation**

 The manuscript has several strengths. First, the authors identify a meaningful gap in previous meta-analyses by focusing on control-group variation as a moderator. This is a valuable contribution because the estimated effectiveness of an educational intervention depends strongly on the nature of the comparison condition. Second, the study attempts to broaden the scope beyond science education by including studies from mathematics, language, social sciences, arts, and culture. Third, the authors use several appropriate quantitative procedures and provide open data, which supports transparency.

 However, the manuscript requires substantial revision before it can be considered methodologically robust. The most important concern is the search strategy. The search string appears too narrow and may have excluded relevant studies using terms such as “guided inquiry,” “scientific inquiry,” “inquiry instruction,” “inquiry approach,” “critical thinking skills,” “higher-order thinking,” or “reasoning skills.” This limitation affects the completeness and representativeness of the systematic review.

 A second major issue concerns the interpretation of the very large pooled effect size. Although the reported effect suggests a strong positive association between inquiry-based learning and critical thinking, the high heterogeneity, significant asymmetry tests, and possible small-study effects mean that the result should be interpreted with caution. The authors should avoid presenting IBL as uniformly effective across all contexts.

 The moderator analyses are interesting but need to be interpreted more carefully. Some subgroups contain only a small number of studies, while several categories were excluded due to insufficient data. This limits the strength of the claims that can be made about country, subject area, educational level, and type of control group. The authors should also define the control-group categories more clearly, especially the distinction between “traditional” and “conventional” instruction, since these terms may overlap in educational research.

 Another important limitation is the absence of a formal risk-of-bias or methodological quality assessment of the included studies. Since the review includes experimental and quasi-experimental studies, study quality may vary substantially. Without such an assessment, it is difficult to know whether the large pooled effect is driven by strong studies or by weaker designs.

 The manuscript also needs language editing. The meaning is generally understandable, but several expressions are grammatically awkward or imprecise. Statistical terminology should also be made more consistent, especially when interpreting Hedges’ g and distinguishing between “large” and “very large” effects.

**Recommendation**

**Major revision**

 The article has a relevant topic and a potentially valuable contribution, especially through its focus on control-group variation in meta-analysis. However, the search strategy, coding procedures, subgroup analyses, publication-bias interpretation, and risk-of-bias assessment need significant strengthening before the manuscript can be accepted.

**Main revisions required**

1. Expand and justify the search strategy using additional keywords and synonyms related to inquiry-based learning and critical thinking.
2. Provide complete search strings for each database, including dates, filters, and field restrictions.
3. Add a formal quality or risk-of-bias assessment of the included studies.
4. Clarify the coding criteria for all moderator variables, especially the distinction between “traditional” and “conventional” control groups.
5. Report whether independent coders were used, how disagreements were resolved, and whether intercoder reliability was calculated.
6. Interpret the very large pooled effect size more cautiously in light of high heterogeneity and possible small-study effects.
7. Revise the publication-bias section to avoid contradictory interpretations.
8. Temper conclusions from subgroup analyses where the number of studies is small.
9. Discuss implementation fidelity, type of inquiry, intervention duration, and assessment instruments as possible sources of heterogeneity.
10. Improve the academic English and consistency of statistical terminology throughout the manuscript.

Are the rationale for, and objectives of, the Systematic Review clearly stated?

Yes

Is the statistical analysis and its interpretation appropriate?

Partly

If this is a Living Systematic Review, is the ‘living’ method appropriate and is the search schedule clearly defined and justified? (‘Living Systematic Review’ or a variation of this term should be included in the title.)

Not applicable

Are sufficient details of the methods and analysis provided to allow replication by others?

Partly

Are the conclusions drawn adequately supported by the results presented in the review?

Partly

Reviewer Expertise:

My research focuses on physics education and science education, with particular interest in inquiry-based learning, students’ misconceptions, scientific literacy, preservice teacher education, artificial intelligence in science teaching, and the use of real-world contexts to support conceptual understanding in physics and science.

I confirm that I have read this submission and believe that I have an appropriate level of expertise to confirm that it is of an acceptable scientific standard, however I have significant reservations, as outlined above.

# Response

**Published:** 2026-05-29

**Authors:** Abel Harapan

Dear Reviewer,

 Thank you for your constructive and detailed feedback on our manuscript. We appreciate your comments regarding the search strategy and the completeness of the systematic review process.

 We would like to seek clarification regarding your recommendation to expand the search strategy using additional keywords and synonyms related to inquiry-based learning and critical thinking.

 Specifically, we would like to confirm whether your suggestion implies that we should:

 1. Conduct a new literature search using the expanded search strings,

 2. Re-screen potentially relevant studies identified through the updated search process, and

 3. Re-run the meta-analysis if additional eligible studies are found.

 Alternatively, would it be sufficient to revise and justify the existing search strategy more comprehensively if the updated search does not identify additional eligible studies?

 We want to ensure that our revision aligns accurately with your expectations and methodological standards.

 Thank you again for your valuable feedback.

 Best regards,

 Author

 [Abel Harapan]

# Response

**Published:** 2026-07-10

**Authors:** Abel Harapan

We sincerely thank for your constructive comments and valuable suggestions. Your feedback has helped us improve the quality, transparency, and rigor of our manuscript. The revisions made in response to your comments are summarized below.

 1. Search strategy

 We expanded the search strings for each database and presented them in a table, including the applied filters, field restrictions, and search dates. As a result, 51 independent studies were included, yielding 57 effect sizes because several studies reported more than one effect size. Accordingly, we re-ran the meta-analysis and revised the results, moderator analyses, and interpretation.

 2. Risk-of-bias assessment

 We added a formal risk-of-bias assessment using the revised JBI Critical Appraisal Checklist for Quasi-Experimental Studies developed by Barker et al. (2024). This tool was selected because most of the included studies employed quasi-experimental designs.

 3. Coding of comparison groups

 Regarding the distinction between “traditional” and “conventional” control groups, we searched the literature for a clear conceptual distinction between these terms but found no consistent differentiation. Therefore, we recoded the comparison groups into broader categories: teacher-centered learning (TCL) and student-centered learning (SCL). Traditional, conventional, and expository instruction were categorized as TCL because these approaches generally position the teacher as the main source of explanation and the primary controller of content, activities, materials, and pacing. In contrast, cooperative learning, problem-based learning, project-based learning, and communicative language teaching were categorized as SCL because these approaches provide more opportunities for active student participation.

 4. Coding procedure and inter-rater reliability

 We added information explaining that the coding process was conducted by two independent coders. Any discrepancies were resolved through discussion. Inter-rater reliability was assessed using Cohen’s kappa.

 5. Interpretation of findings

 We revised the interpretation of the main effect, heterogeneity, subgroup analyses, small-study effects, and publication bias more cautiously.

 5. Additional potential moderators

 We discussed the potential moderators recommended by the reviewer, including implementation fidelity, type of inquiry, intervention duration, and assessment instruments. These variables were discussed in relation to previous relevant meta-analyses that examined whether they significantly moderated the effect of inquiry-based learning.

 We also updated the extended materials by adding evidence of inter-rater coding reliability, the risk-of-bias assessment, the sensitivity analysis, and the R script used to calculate Cohen’s kappa. In addition, we clarified the coding procedures and revised the interpretation of heterogeneity, publication bias, small-study effects, and moderator analyses. We hope these revisions have improved the transparency, reproducibility, and methodological rigor of the manuscript.
